# Evidence for the Introduction, Reassortment, and Persistence of Diverse Influenza A Viruses in Antarctica

**DOI:** 10.1128/JVI.01404-16

**Published:** 2016-10-14

**Authors:** Aeron C. Hurt, Yvonne C. F. Su, Malet Aban, Heidi Peck, Hilda Lau, Chantal Baas, Yi-Mo Deng, Natalie Spirason, Patrik Ellström, Jorge Hernandez, Bjorn Olsen, Ian G. Barr, Dhanasekaran Vijaykrishna, Daniel Gonzalez-Acuna

**Affiliations:** a WHO Collaborating Centre for Reference and Research on Influenza, Parkville, Victoria, Australia; b University of Melbourne, Melbourne School of Population and Global Health, Parkville, Victoria, Australia; c Program in Emerging Infectious Diseases, Duke-NUS Medical School, Singapore; d Zoonosis Science Center, IMBIM, Uppsala University, Uppsala, Sweden; e Department of Microbiology, Kalmar County Hospital, Kalmar, Sweden; f Medical Sciences, Uppsala University, Uppsala, Sweden; g Universidad de Concepción, Facultad de Ciencias Veterinarias, Chillán, Chile; St. Jude Children's Research Hospital

## Abstract

Avian influenza virus (AIV) surveillance in Antarctica during 2013 revealed the prevalence of evolutionarily distinct influenza viruses of the H11N2 subtype in Adélie penguins. Here we present results from the continued surveillance of AIV on the Antarctic Peninsula during 2014 and 2015. In addition to the continued detection of H11 subtype viruses in a snowy sheathbill during 2014, we isolated a novel H5N5 subtype virus from a chinstrap penguin during 2015. Gene sequencing and phylogenetic analysis revealed that the H11 virus detected in 2014 had a >99.1% nucleotide similarity to the H11N2 viruses isolated in 2013, suggesting the continued prevalence of this virus in Antarctica over multiple years. However, phylogenetic analysis of the H5N5 virus showed that the genome segments were recently introduced to the continent, except for the NP gene, which was similar to that in the endemic H11N2 viruses. Our analysis indicates geographically diverse origins for the H5N5 virus genes, with the majority of its genome segments derived from North American lineage viruses but the neuraminidase gene derived from a Eurasian lineage virus. In summary, we show the persistence of AIV lineages in Antarctica over multiple years, the recent introduction of gene segments from diverse regions, and reassortment between different AIV lineages in Antarctica, which together significantly increase our understanding of AIV ecology in this fragile and pristine environment.

**IMPORTANCE** Analysis of avian influenza viruses (AIVs) detected in Antarctica reveals both the relatively recent introduction of an H5N5 AIV, predominantly of North American-like origin, and the persistence of an evolutionarily divergent H11 AIV. These data demonstrate that the flow of viruses from North America may be more common than initially thought and that, once introduced, these AIVs have the potential to be maintained within Antarctica. The future introduction of AIVs from North America into the Antarctic Peninsula is of particular concern given that highly pathogenic H5Nx viruses have recently been circulating among wild birds in parts of Canada and the Unites States following the movement of these viruses from Eurasia via migratory birds. The introduction of a highly pathogenic influenza virus in penguin colonies within Antarctica might have devastating consequences.

## INTRODUCTION

Aquatic birds are the natural reservoir for influenza A viruses, and their ecology and migratory patterns have direct impacts on the spread and diversity of avian influenza viruses (AIVs) across the globe (1). Migratory birds spread AIVs through local movements or across larger distances, commonly along established intercontinental flyways (1). Although there are locations of crossover between these global flyways, there is a clear genetic distinction between AIVs detected in spatially segregated birds of the American and Eurasian landmasses, resulting in the divergence of AIV lineages into the American and Eurasian AIV lineages (1). However, most of our understanding of AIV ecology is based on surveillance conducted in the Northern Hemisphere, with substantially less AIV genetic data from the Southern Hemisphere.

The major migratory bird flyways extend from the North American and Eurasian regions through continents in the Southern Hemisphere, resulting in the close relationship of viruses in the Americas, whereas the African and Australian AIVs show greater similarity to Eurasian viruses. Recently, increased surveillance in Australia has shown that while new strains are frequently introduced from Europe or Asia, the endemicity of imported viruses over time produces a group of viruses that are genetically distinct from those circulating in the northern landmasses (2, 3). A similar evolutionary divergence has been observed in some South American (4, 5) and African (6) AIVs, although publically available AIV sequence data are limited for both of these continents.

In 2013, we reported the first detection and isolation of an AIV from Adélie penguins in Antarctica, the most southern and isolated continent on the planet (7). Consistent with the theory that the evolutionary divergence of AIVs increases with further advancement south, we described an H11N2 virus that had diverged from other AIVs between 49 and 80 years ago (7). Although earlier AIV studies in Antarctica had detected influenza A virus antibodies in penguins and other birds (8), to date, the H11N2 virus remains our only insight into AIVs in Antarctica. As such, we hypothesized that Antarctica is the ultimate “AIV evolutionary sink,” whereby new strains are introduced into the region only on rare occasions but, once introduced, can become endemic within the local bird population and over time become highly diverged from other AIVs on the planet. However, as a result of continued AIV surveillance in penguins and other birds in 2014 and 2015, we are able to report the successful detection of additional AIVs in Antarctica. The genetic analysis of these viruses has further increased our understanding of the ecology of AIVs by demonstrating the local persistence of the H11 AIV detected previously but also the recent introduction of a novel H5N5 reassortant AIV, whose genes were derived from both the American and Eurasian lineages, into the continent.

## MATERIALS AND METHODS

### Surveillance and sample collection.

We conducted AIV surveillance during 2014 and 2015 in the following five locations on the Antarctic Peninsula (see Fig. S1 in the supplemental material): Kopaitik Island, Rada Covadonga (63°19′S, 57°51′W), in both 2014 and 2015; Neko Harbor, Anvord Bay (64°50′S, 62°33′W), and Gabriel Gonzalez Videla Base, Paradise Bay (64°49′S, 62°51′W), in 2014; and Cape Shirreff, Livingston Island, (62°27′S, 60°47′W), and Narebski Point, King George Island (62°14′S, 58°46′W), in 2015. Between 28 January and 9 February 2014, cloacal swabs were taken from three species of birds, namely, gentoo penguin (Pygoscelis papua), brown skua (Stercorarius antarcticus), and snowy sheathbill (Chionis albus), and between 8 January and 8 February 2015, combined cloacal and tracheal swabs were taken from chinstrap penguins (Pygoscelis antarcticus). In addition to the swabs, fresh fecal samples were taken from the ground within gentoo penguin colonies and next to brown skuas and snowy sheathbills in 2014. Blood was collected from 307 of the chinstrap penguins that were swabbed in 2015.

Cloacal, tracheal, or environmental swabs were placed in tubes containing viral transport medium (brain heart infusion [BHI] broth-based medium [Oxoid] with 0.3 mg/ml penicillin, 5 mg/ml streptomycin, 0.1 mg/ml gentamicin, and 2.5 μg/ml amphotericin B). Swab specimens were kept on ice for up to 4 h before being frozen at −80°C. Blood was collected from chinstrap penguins, from either wing or foot veins, placed into serum clot activating tubes, and centrifuged, and sera were stored at either −80°C or −20°C. Approvals to conduct sampling from penguins in Antarctica were provided by the Universidad de Concepción, Facultad de Ciencias Veterinarias, Chillán, Chile (application CBE-48-2013), and the Instituto Antártico Chileno, Chile (application 654).

### RT-PCR and sequence analysis.

RNAs were isolated from swabs by use of the QIAextractor system (Qiagen, Australia). All samples were tested by a one-step reverse transcription-PCR (RT-PCR) assay targeting the influenza virus matrix genome segment as designed by the CDC (9), using a SensiFast Probe Lo-ROX One-Step kit (Bioline, Australia) on an Applied Biosystems 7500 Fast Real Time PCR system (Life Technologies, Australia). Samples with a cycle threshold (*C_T_*) value of <42 cycles or cultured influenza viruses were analyzed further by conventional RT-PCR, using universal primers targeting each of the eight influenza virus gene segments (10). Amplified RT-PCR products were sequenced on an Applied Biosystems 3500XL genetic analyzer as previously described (11) or by next-generation sequencing (NGS). For NGS, a 400-bp DNA library was generated by fragmentation followed by Ion Xpress Barcode Adapter ligation, using an Ion Xpress Plus fragment library kit (Thermo Fisher Scientific) according to the manufacturer's instructions. Emulsion PCR was performed on an Ion Touch 2 machine, and the final product was cleaned and enriched for template-positive ion sphere particles on an Ion One Touch ES instrument (Thermo Fisher Scientific). An Ion 314v2 chip was used for sequencing on an Ion Torrent PGM instrument (Thermo Fisher Scientific). *De novo* analysis of the NGS data was first performed using CLC Genomic Benchtop 7 (Qiagen). All the resulting contigs were then subjected to BLAST searches to find influenza virus-related sequences. The closest-matched complete influenza virus segment sequences were chosen as reference sequences for a second round of analysis to map all the reads to generate final influenza virus segment sequences.

### Virus isolation.

Virus isolation from RT-PCR-positive samples was attempted by inoculating the swab specimen (diluted 1:1 with phosphate-buffered saline [PBS] containing a 1% neomycin-polymyxin solution [bioCSL, Australia]) into the allantoic cavity of 11-day-old embryonated hens' eggs. Allantoic fluid was harvested after 3 days, and influenza virus was detected by hemagglutination with turkey erythrocytes. The 50% egg infective dose (EID_50_) per milliliter was determined for each isolate by performing log_10_ dilutions in PBS containing a 1% neomycin-polymyxin solution and infecting the allantoic cavity of 11-day-old embryonated hens' eggs.

### Phylogenetic and dating analyses.

To identify the phylogenetic relationships of the AIVs detected in this study, we downloaded the sequences of all avian H5 and N5 genes and six internal genes (PB2, PB1, PA, NP, MP, and NS) of all other AIV subtypes (>6,000 global avian sequences) that were available from the GenBank and GISAID databases on 1 April 2016. All alignments were performed and checked manually in Geneious, version 9.0.3 (Biomatters Ltd., New Zealand). In addition, sequences from equine H3N8 viruses, the H11N2 virus from an Antarctic penguin in 2013, and representative swine and human viruses were included in the analysis. The final H5 and N5 data sets comprised 1,370 and 450 sequences, respectively, whereas the six internal gene data sets were randomly downsampled to 2,102 sequences. For each gene segment, maximum likelihood (ML) analysis was performed with RAxML v.8.0.14 (12) and by using the general time-reversible (GTR) + Γ nucleotide substitution model.

Based on the ML gene topologies, we further selected the lineages of interest to which the penguin H5N5 subtype belonged to and used the corresponding lineages to estimate the divergence times of the novel virus. For each segment, a coalescent-based approach was employed by using a Gaussian Markov random field (GMRF) Skyride plot as implemented in BEAST v1.8.2 (13). The uncorrelated lognormal relaxed molecular clock, the HKY85 substitution model, and the SRD06 codon position model (to allow codon partitioning of the 1 plus 2 and 3 positions) were used. At least 4 independent analyses of 100 million generations were performed, with sampling every 10,000 generations. The burn-in values, convergence, and adequate effective sample size (ESS) were checked using Tracer v.1.6. Maximum clade credibility (MCC) trees were subsequently generated from TreeAnnotator.

### Serological analysis.

To test serum samples for broadly reactive influenza virus nucleoprotein (NP) antibodies, a competitive enzyme-linked immunosorbent assay (ELISA) with plates coated with NP was used (AI Ab test; IDEXX, ME). NP antibody-positive sera were further tested for hemagglutinin (HA) antibodies specific to the H11N2 virus (using the isolate A/Adélie Penguin/Antarctica/270/2013) or the H5N5 virus (using the isolate A/Chinstrap penguin/Antarctica/15459/2015) by using a hemagglutination inhibition (HI) assay as previously described (14). Turkey erythrocytes were used for the HI assays, as they yielded 2-fold higher hemagglutination titers for the H11 and H5 viruses than those obtained with chicken erythrocytes. NP antibody-positive sera were also tested in an HI assay for reactivity with the following other AIVs: A/Turnstone/King Island/7040CP/2014 (H3N5), A/Red-necked Stint/Australia/1/2004 (H4N8), A/Gyr Falcon/Washington/41088-6/2014 (H5N8), A/Turnstone/King Island/7019CP/2014 (H6N8), A/Mallard/Netherlands/4/2010 (H7N3), A/Turkey/NSW/F655H9/2012 (H9N2), A/Turnstone/King Island/7109CP/2014 (H10N8), and A/Sharp-tailed Sandpiper/Australia/6/2004 (H11N9).

### Accession number(s).

The following sequences were deposited in the GISAID database (http://www.gisaid.org): H5N5 influenza virus full genome (GISAID isolate ID EPI_ISL_224756; sequence accession numbers EPI 774530, EPI 774531, EPI 774533 to EPI 774536, EPI 774538, and EPI 774539) and H11 influenza virus HA, MP, and NS gene segments (GISAID isolate ID EPI_ISL_224755; sequence accession numbers EPI 774527 to EPI 774529).

## RESULTS

### Detection of influenza viruses.

During 2014, we took 209 cloacal swabs and 86 fresh fecal samples from three bird species: gentoo penguin, brown skua, and snowy sheathbill (Table 1). Of those 295 samples, only one, a cloacal swab from a snowy sheathbill, was found to contain influenza virus RNA following a real-time RT-PCR assay for the influenza virus matrix gene (*C_T_* value of 29.9) (Table 1). The virus, designated A/Snowy sheathbill/Antarctica/2899/2014, was collected on Kopaitik Island and was one of only four samples (two cloacal swabs and two fresh fecal samples) collected from snowy sheathbills (Table 1). The AIV RT-PCR-positive specimen was inoculated into embryonated hens' eggs, but the virus was not successfully cultured, and therefore sequence analysis of the AIV was conducted directly from the specimen. Presumably due to sample degradation or low viral copy numbers, we were able to derive reliable sequence data only for the HA, MP, and NS gene segments. BLAST analysis revealed that the virus was of the H11 subtype.

**TABLE 1:** Numbers of influenza virus RT-PCR-positive specimens from different bird species following collection at various locations of the Antarctic Peninsula in 2014 and 2015

Bird species	Type of sample	No. of influenza virus-positive samples/no. of samples tested^*a*^	
		2014 (28 January to 9 February)	2015 (8 January to 8 February)
Gentoo penguin (Pygoscelis papua)	Cloacal swab	0/195^*b*^	NC
	Fresh fecal sample	0/81	NC
Brown skua (Stercorarius antarcticus)	Cloacal swab	0/12	NC
	Fresh fecal sample	0/3	NC
Snowy sheathbill (Chionis albus)	Cloacal swab	1/2^*c*^	NC
	Fresh fecal sample	0/2	NC
Chinstrap penguin (Pygoscelis antarcticus)	Cloacal/tracheal swab	NC	1/493^*d*^
Total		1/295	1/493

a NC, not collected.

b Included swabs from 189 adult and 6 juvenile birds.

c A(H11) influenza virus detected and designated A/Snowy sheathbill/Antarctica/2899/2014.

d A(H5N5) influenza virus detected and designated A/Chinstrap penguin/Antarctica/15459/2015.

In 2015, combined cloacal/tracheal swabs were taken from 493 chinstrap penguins, among which one was found to contain influenza virus RNA, with a *C_T_* value of 29.7 for an RT-PCR assay of the influenza virus matrix gene (Table 1). The virus was designated A/Chinstrap penguin/Antarctica/15459/2015 and was found in 1 of 161 swabs collected from penguins on Narebski Point, King George Island. The chinstrap penguins from the other two locations (Kopaitik Island [*n* = 169] and Cape Shirreff [*n* = 163]) were AIV negative. The AIV-positive specimen was successfully cultured in embryonated hens' eggs, to an HA titer of 256/25 μl, and sequence analysis of the virus isolate revealed an H5N5 strain that did not contain a multibasic cleavage site in the HA.

### Detection of influenza virus antibodies.

Sera from 307 chinstrap penguins were collected in 2015. The frequency of NP antibody-positive penguins from Narebski Point, King George Island (the location where the H5N5 strain was detected in 2015), was 31% (31/100 penguins), compared to 14% (14/103 penguins) for penguins from Kopaitik Island (where the H11 viruses were detected in 2013 [7] and 2014 [present study]) and 10% (10/104 penguins) for penguins from Cape Shirreff. Serum was not taken from the chinstrap penguin at Narebski Point that was shedding the H5N5 AIV. The 55 NP antibody-positive sera were tested by hemagglutination inhibition (HI) assay for specific antibodies to the H11N2 egg-propagated virus detected in the Adélie penguins in 2013 (7) or the H5N5 egg-propagated virus detected in a chinstrap penguin in this study. Interestingly, none of the sera had any HI reactivity with the H5N5 virus, and only three serum samples reacted with the H11N2 virus: two from Narebski Point (with titers of 40 and 320) and one from Kopaitik Island, where H11 viruses were detected in 2013 (7) and 2014 (present study). None of the NP antibody-positive sera reacted with a selection of AIVs (H3, H4, H5, H6, H7, H9, H10, and H11) isolated outside Antarctica. The three serum samples which reacted with the H11N2 A/Adélie Penguin/Antarctica/270/2013 virus did not react with the H11N9 A/Sharp-tailed Sandpiper/Australia/6/2004 virus, demonstrating a significant antigenic difference between these H11 viruses.

### Phylogenetic relationships and divergence dates of Antarctic influenza viruses.

The three gene segments successfully sequenced from the H11 virus A/Snowy sheathbill/Antarctica/2899/2014 were nearly identical (>99.7%, 99.8%, and 99.1% nucleotide sequence similarities for the HA, MP, and NS segments, respectively) to those of the H11N2 viruses detected in Adélie penguins (e.g., A/Adélie Penguin/Antarctica/226/2013) at the same location (Kopaitik Island) approximately 12 months earlier (7). As such, the phylogenetic positions of the HA, MP, and NS segments of the A/Snowy sheathbill/Antarctica/2899/2014 virus were highly diverged from those of other AIVs, as observed previously for the A/Adélie Penguin/Antarctica/226/2013 H11N2 virus. The HA and NS genes were most closely related to North American avian lineage viruses from the 1970s to 1980s (see Fig. S2 and S3 in the supplemental material), while the MP gene formed a close relationship with North American viruses (1974 to 2001) but appeared to be the closest ancestor to a large number of South American AIVs (see Fig. S4).

The continuous detection of genetically similar H11N2 viruses over 2 years in the same location in Antarctica, despite the absence of hosts during long periods of nonsummer months, raised the possibility of the persistence of the virus in frozen ice. To test this hypothesis, we compared the nucleotide substitution rates of all gene segments of the Antarctic H11N2 viruses with those of their global counterparts (Table 2). Considerably lower evolutionary rates were observed for the PB2, HA, NP, and NA genes of the Antarctic H11N2 viruses than for the global data set, whereas marginally lower rates were observed for the PB1 and MP genes. In contrast, the rates for the PA and NS genes were similar. The consistently lower evolutionary rates for the Antarctic H11N2 viruses for most segments support the hypothesis of viruses reemerging after being frozen in ice. Furthermore, the segments that exhibited similar rates may have recently been introduced into the ecosystem through reassortment. However, a thorough investigation with virological data from Antarctica over several years is necessary to better understand the dynamics of the reemergence and evolutionary rate differences that we observed in the H11N2 viruses.

**TABLE 2:** Nucleotide substitution rates of globally collected H11N2 viruses and 2013-2014 H11N2 viruses from Antarctica^*a*^

Segment	Nucleotide substitution rate (10^−3^) of H11N2 viruses					
	Globally collected viruses			Antarctic viruses		
	Mean	95% lower HPD	95% upper HPD	Mean	95% lower HPD	95% upper HPD
PB2	2.44	2.29	2.59	1.1	0.8	1.3
PB1	1.71	1.47	1.94	1.4	1.0	1.9
PA	2.26	1.97	2.56	2.3	1.5	3.2
HA	2.9	2.3	3.5	1.2	0.9	1.6
NP	2.07	1.89	2.24	1.0	0.7	1.3
NA	2.8	2.4	3.1	1.8	1.4	2.2
MP	1.72	1.45	2.01	1.4	0.6	2.3
NS	1.47	1.30	1.65	1.5	0.9	2.2

a HPD, highest posterior density.

**FIG 1:**
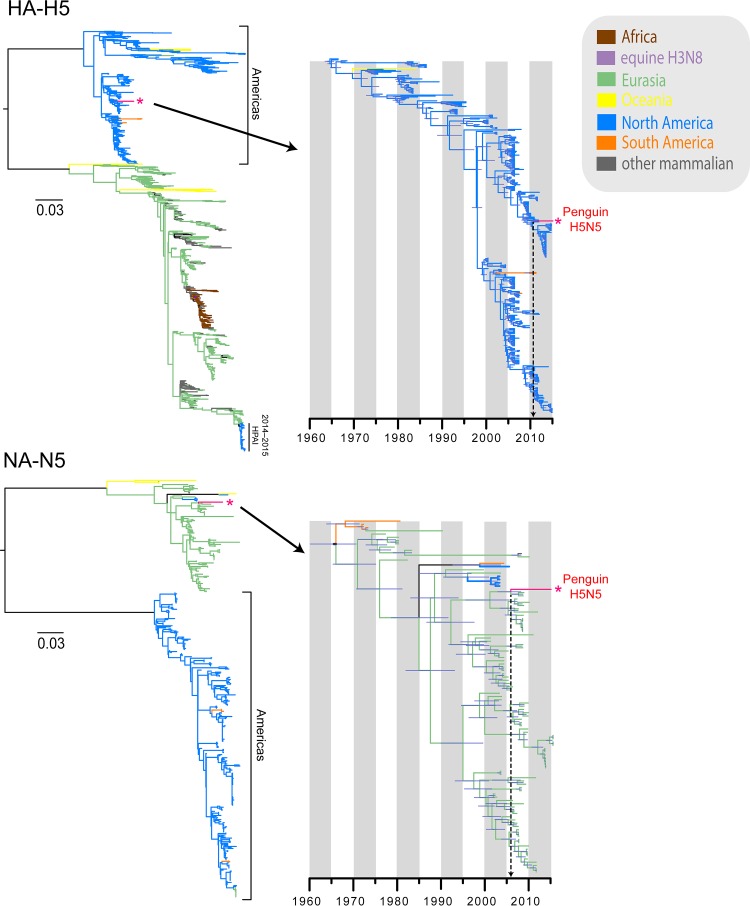
Maximum likelihood phylogenies (left) and divergence times (right) of H5-HA and N5-NA segments. Different branch colors denote different geographical regions and different subtypes; note that blue branches represent North American avian lineages, whereas light green branches indicate Eurasian avian lineages. The red asterisks indicate the novel 2015 penguin H5N5 virus.

**FIG 2:**
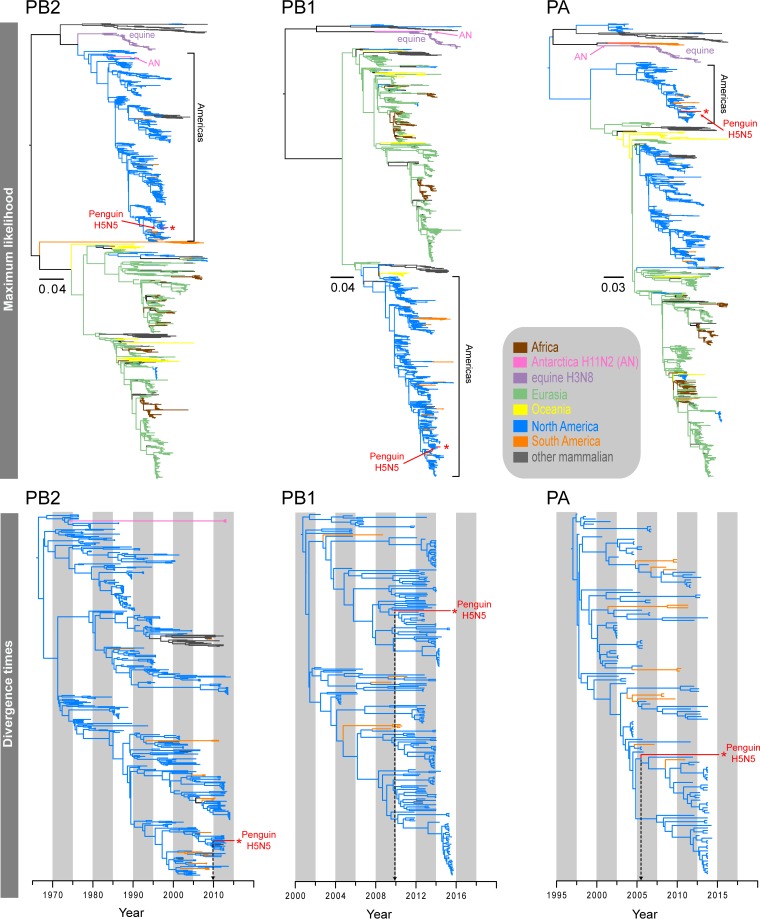
Maximum likelihood phylogenies (top) and divergence times (bottom) of the PB2, PB1, and PA genes. Different branch colors denote different geographical regions and different subtypes; note that blue branches represent North American avian lineages, whereas light green branches indicate Eurasian avian lineages. The red asterisks indicate the novel 2015 penguin H5N5 virus.

**FIG 3:**
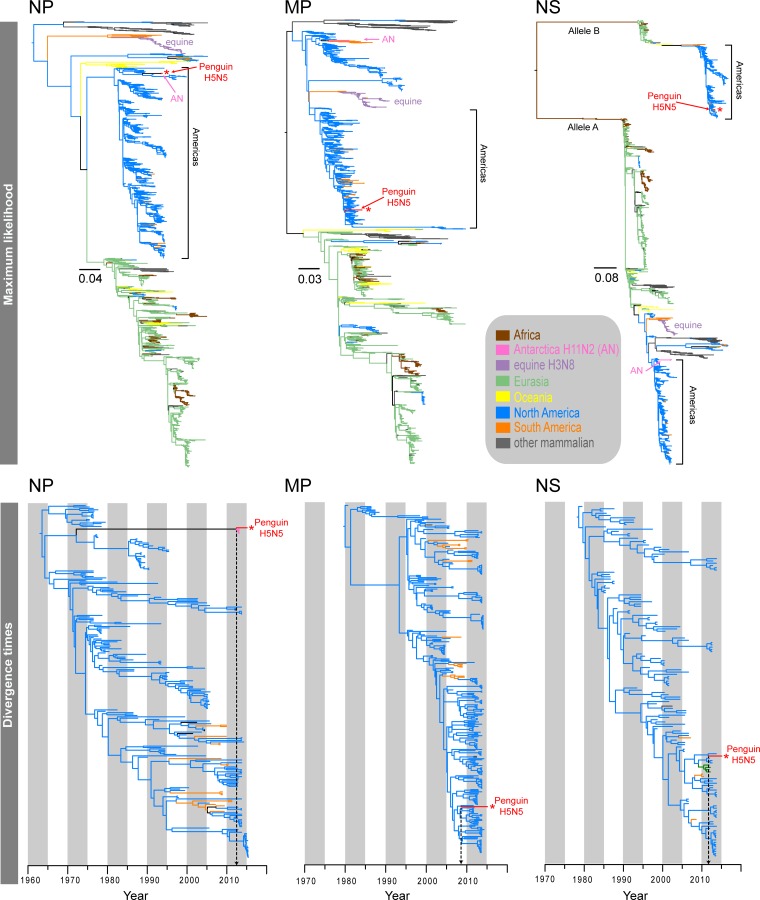
Maximum likelihood phylogenies (top) and divergence times (bottom) of the NP, MP, and NS genes. Different branch colors denote different geographical regions and different subtypes; note that blue branches represent North American avian lineages, whereas light green branches indicate Eurasian avian lineages. The red asterisks indicate the novel 2015 penguin H5N5 virus.

In contrast to the highly divergent H11 virus, the gene constellation of the novel H5N5 virus A/Chinstrap penguin/Antarctica/15459/2015 was substantially less diverged from those of AIVs circulating elsewhere in the world. The H5N5 virus possessed seven gene segments (PB2, PB1, PA, HA, NP, MP, and NS) that were derived from the North American avian lineages (blue branches in Fig. 1 to 3; see Fig. S5 and S7 to S12 in the supplemental material), with the exception of the NA-N5 segment, which unequivocally originated from the Eurasian avian lineages (pale green branches in Fig. 1; see Fig. S6). We found that the H5 segment from the Antarctic virus is closely related to contemporary low-pathogenicity avian influenza (LPAI) H5 viruses from North America (see Fig. S5), as found in numerous wild birds, such as northern shovelers, mallards, blue-winged teals, and American wigeons, whereas the NA-N5 segment is mostly closely related to those of viruses isolated from wild ducks in Korea and Vietnam in 2008 and 2009 (see Fig. S6). The detection of the Eurasian-derived NA-N5 lineage is surprising, as this NA lineage has not previously been reported from the Americas.

The six internal gene segments of the H5N5 virus clustered within the North American avian lineages (Fig. 2 and 3). Interestingly, both the Antarctic H5N5 and H11N2 viruses shared the same ancestor for the NP segment, exclusively forming a strongly supported monophyletic clade (posterior probability [PP] = 1.00), whereas the remaining internal segments displayed marked differences in the phylogenetic placements of the H5N5 and H11N2 viruses detected in Antarctica. The PB1 and PA segments of the H5N5 virus were well nested within the North American avian influenza virus lineages (blue branches in Fig. 3), whereas the H11N2 segments were sister to the divergent North American equine H3N8 viruses (purple branches in Fig. 3). The PB2 segments of both H5N5 and H11N2 viruses belonged to the same avian lineage; however, the H5N5 PB2 segment was closely related to those of recent avian strains (2011 to 2013), whereas the H11N2 PB2 segment was most closely related to older strains collected in 1976 (see Fig. S12 in the supplemental material). The MP and NS segments of H5N5 and H11N2 viruses belonged to two distinct evolutionary avian lineages. The H5N5 MP segment was clearly clustered within the North American avian lineage (see Fig. S8), whereas the H11N2 MP segment formed a sister group with the South American avian lineage (orange branches in Fig. 3). The NS segments of the H11N2 and H5N5 viruses were clearly distinct, as they belonged to NS alleles A and B, respectively (Fig. 3). More specifically, the H5N5 NS segment appeared to be closely related to those of viruses from North American mallards but also clustered within a well-supported lineage (PP = 0.99) (see Fig. S17) comprised of both Eurasian wild waterfowl H9N2 viruses (i.e., from Dongting Lake in China in 2011 and 2012) and South American wild bird H6N8 viruses detected in 2010. This evidence suggests that the H5N5 virus from the chinstrap penguin is most likely the result of reassortment of gene segments originating from diverse avian sources.

**FIG 4:**
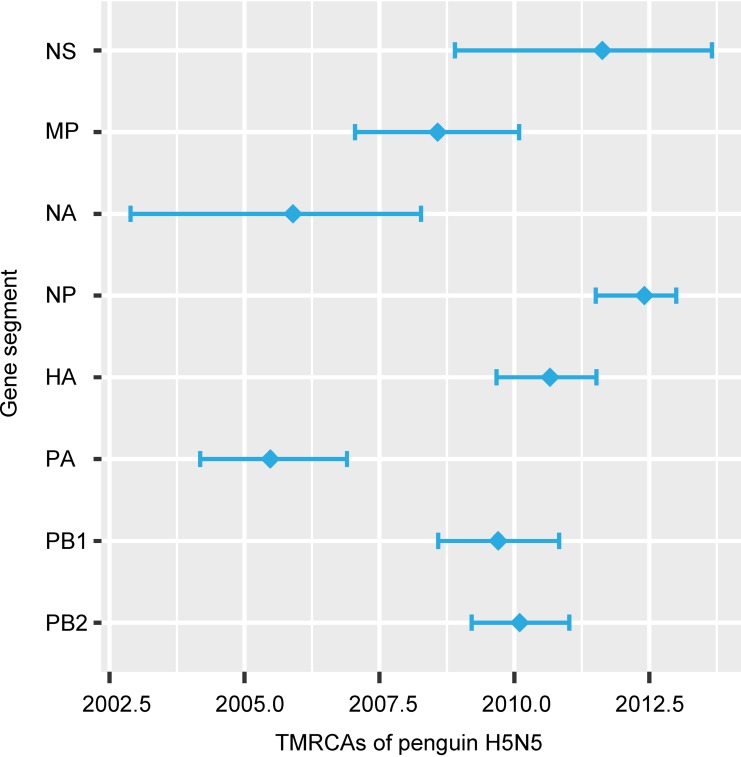
TMRCA estimates for each gene segment of the 2015 penguin H5N5 virus from Antarctica. Diamonds indicate mean TMRCAs, and horizontal bars denote the 95% HPD intervals.

To identify the timeline of reassortment and origins of the Antarctic H5N5 virus segments, we estimated the divergence times of all gene segments by using the Bayesian Markov chain Monte Carlo (MCMC) relaxed clock approach. Our temporal phylogenies (Fig. 4; Table 3) demonstrated marked variations in the mean time of the most recent common ancestor (TMRCA) for individual segments, ranging from June 2005 (PA segment) (highest posterior density [HPD], March 2004 to November 2006) to May 2012 (NP segment) (HPD, July 2011 to January 2013). The mean TMRCA for the lineages containing the Antarctic H5 lineages and the most closely related North American H5 segments was August 2010 (HPD, September 2009 to July 2011), whereas the mean TMRCA for the Antarctic N5 and Eurasian N5 lineages was November 2005 (HPD, November 2002 to April 2008). These recent divergence times are substantially different from the long divergence times observed for the H11 viruses reported previously (7) and in the present study.

**TABLE 3:** Divergence times of genes of the chinstrap penguin H5N5 virus collected in Antarctica

Segment	Time to most recent common ancestor^*a*^			Nucleotide substitution rate (10^−3^)^*b*^		
	Mean	95% lower HPD	95% upper HPD	Mean	95% lower HPD	95% upper HPD
PB2	February 2010	March 2009	January 2011	2.44	2.89	2.59
PB1	September 2009	August 2008	October 2010	2.36	2.07	2.65
PA	June 2005	March 2004	November 2006	2.32	2.06	2.57
HA	August 2010	September 2009	July 2011	3.23	2.95	3.52
NP	May 2012	July 2011	January 2013	2.07	1.89	2.24
NA	November 2005	November 2002	April 2008	2.56	2.05	3.05
MP	August 2008	January 2007	February 2010	1.54	1.32	1.78
NS	August 2011	November 2008	August 2013	1.37	1.12	1.61

a TMRCAs for the chinstrap penguin H5N5 virus and the most closely related virus clade, representing the period of divergence and unsampled diversity of this lineage.

b Estimated for the H5N5 data sets shown in Fig. S13 to S20 in the supplemental material.

## DISCUSSION

Understanding the ecology of AIVs in Antarctica is important for furthering our understanding of the global movement of these viruses and to help in assessing the risk that highly pathogenic (HP) viruses will be introduced into this unique natural environment. Overall, the frequency of AIV detection across 3 years of samples (2013 to 2015) remained low (10/1,089 samples [0.9%]), with no AIVs detected in gentoo penguins in 2014, a 0.2% AIV prevalence in chinstrap penguins in 2015, and a 2.7% AIV prevalence in Adélie penguins in 2013 (7). It is unclear what factors may explain these year-to-year differences in AIV frequency or whether some species, such as the Adélie penguin, are more susceptible to AIV than others. Serological analysis of sera from chinstrap penguins showed an overall frequency of AIV NP antibodies of 17.9% (55/307 samples), in comparison to 15.9% (43/270 samples) for adult and juvenile Adélie penguins in 2013 and 22.1% (43/195 samples) for only adult Adélie penguins (7). These serologic data are comparable to those from other studies of penguins that showed AIV antibody prevalences of 11.8 to 12.5% in gentoo penguins (15, 16), 3.7 to 58.4% in Adélie penguins (8, 16, 17), and 10.8% in chinstrap penguins (16), although several other studies failed to detect any influenza virus antibodies in these or other penguin species in Antarctica (8, 17, 18). As we found in our previous study, only a small proportion of the NP-positive sera reacted with the H11N2 virus in an HI assay (7), and none had H5N5-specific antibodies, suggesting either prior infection with an AIV other than the H11 or H5 virus or a more rapid waning of HA antibody titers than those of NP antibodies.

The detection of an AIV in a snowy sheathbill demonstrates the potential for nonpenguin bird species in Antarctica to also become infected and potentially to aid in the spread of AIVs. The snowy sheathbill is a scavenging bird that has a close interaction with penguin colonies, commonly stealing krill and fish from penguins and feeding on young chicks as well as animal feces. Occasionally, some snowy sheathbills may travel further north to the Falkland Islands and the most southern coastal regions of South America (19), and therefore they may facilitate the movement of AIVs between mainland South America and the Antarctic Peninsula.

The finding of an H11 virus that, based on sequencing of three gene segments, is nearly identical to the H11N2 virus found approximately 12 months earlier in the same location suggests the persistence of this strain in a localized region. Kopaitik Island is densely inhabited by birds during the summer period (November to February), but during the nonsummer months birds move out onto the pack ice, where the air temperature is higher than that on land and where they can find cracks in the ice to catch fish. While it is possible that AIV infections may be maintained among birds on the pack ice during the winter period and then brought back to the breeding grounds, the birds are more spread out, making transmission less likely than it is during the summer period. An alternative possibility for local AIV persistence is preservation in the ice at the breeding sites over winter, with AIVs then becoming available for the infection of new susceptible penguins in the breeding colonies when the ice thaws. AIVs have been shown experimentally to exhibit considerable viable persistence in frozen (−20°C and −30°C) fresh, brackish, or salty water for up to 12 months, even during multiple freeze-thaw cycles (20). Although local AIV persistence may occur, it is also possible that the H11N2 virus and other AIVs move regularly between the Antarctic Peninsula and regions of South America but that we are unaware of this movement because of undersampling.

The detection of an H5N5 virus in a chinstrap penguin in 2015 which, apart from the NA segment, was closely related to recently circulating viruses in North America demonstrates a relatively recent introduction of an AIV from an avian source that subsequently circulated for at least 8 years (approximately between 2005 and 2012) prior to our detection. Our data suggest that independent introduction events from diverse origins may have occurred, probably via the avian Pacific-Americas flyway from North America down to South America and then to the northern tip of the Antarctica Peninsula. Although the migratory paths of many species in this flyway are not thought to extend to Antarctica, a small number of bird species, including the Arctic tern (Sterna paradisaea) (21) and the south polar skua (Stercorarius maccormicki) (22), do conduct transhemispheric migrations to Antarctica and therefore may introduce AIVs into the continent. We speculate that viruses carrying the Eurasian NA-N5 segment may have been introduced sporadically into North America from Eurasia by migratory birds using the East Asian-Australasian migration flyway (possibly via stopovers in Iceland and Alaska). However, viruses with a Eurasian N5 segment have not previously been reported in the Americas, so these viruses either were present at low frequencies or were present but are no longer, or the reassortment of the NA segment of the H5N5 virus into a predominantly North American-like virus may have occurred not in the Americas but instead in Antarctica.

In summary, the results of this study expand our knowledge of the ecology of AIVs on the Antarctic Peninsula. While providing clear evidence for the local persistence of the H11 AIVs in the same location, our detection of an H5N5 virus demonstrates that contemporary AIVs are being introduced into the region from North America. This finding is particularly concerning given the recent emergence of highly pathogenic AIVs (HPAIVs) belonging to the H5 clade 2.3.4.4 in North America in late 2014 (23). Importantly, many of these viruses were detected in apparently healthy wild waterfowl, thereby potentially facilitating the spread of the virus. Fortunately, these H5 HPAIVs have apparently disappeared from North America (24), but any future outbreak of HPAIVs in wild birds in either North or South America may potentially have an impact in the Antarctic Peninsula, particularly if viruses are readily spread by key species that migrate to that region. As such, continued AIV surveillance in Antarctica, together with a high level of vigilance of the disease status of the diverse group of birds that are native to the region, remains important.

## Supplementary Material

Supplemental material
