# Supplementary material for: Evidence for the Introduction, Reassortment, and Persistence of Diverse Influenza A Viruses in Antarctica
Source: J Virol. 2016 Oct 14;90(21):9674–82. doi: 10.1128/JVI.01404-16 (PMC5068520; doi:10.1128/JVI.01404-16)
Supplement: Supplemental material [file supp_90_21_9674__index.html]

Supplemental material 

# Evidence for the Introduction, Reassortment, and Persistence of Diverse Influenza A Viruses in Antarctica

## Supplemental material

- Supplemental file 1 -

  Fig. S1 (Map of Antarctica and sampling sites on the Antarctic Peninsula.)

  Fig. S2 to S4 (Maximum-likelihood evolutionary trees of various sequences showing phylogenetic similarity of the H11 virus A/Snowy sheathbill/Antarctica/2899/2014 and the H11N2 viruses detected in Adélie penguins in 2013.)

  Fig. S5 to S12 (Maximum-likelihood evolutionary tree of various sequences from avian, equine, human and swine viruses.)

  Fig. S13 to S20 (Dated evolutionary trees of various sequences from avian, equine, human and swine viruses.)

  PDF, 3.8M
