# Supplementary material for: Evidence for the Introduction, Reassortment, and Persistence of Diverse Influenza A Viruses in Antarctica
Source: J Virol. 2016 Oct 14;90(21):9674–82. doi: 10.1128/JVI.01404-16 (PMC5068520; doi:10.1128/JVI.01404-16)
Supplement: Supplemental material [file JVI.01404-16_zjv999182045so1.pdf]

Supplementary data – Hurt et al. Evidence for the introduction, reassortment and persistence of diverse influenza A viruses in Antarctica

Supplementary Figure legends

Supplementary Figure 1. Map of Antarctica and sampling sites on the Antarctic Peninsula.

Supplementary Figure 2. Maximum-likelihood evolutionary tree of H11-HA sequences showing phylogenetic similarity of the H11 virus, A/Snowy sheathbill/Antarctica/2899/2014 (orange branch), detected in 2014, with the H11N2 viruses detected in Adélie penguins in 2013 (red branches).

Supplementary Figure 3. Maximum-likelihood evolutionary tree of MP sequences showing phylogenetic similarity of the H11 virus, A/Snowy sheathbill/Antarctica/2899/2014 (orange branch), detected in 2014, with the H11N2 viruses detected in Adélie penguins in 2013 (red branches).

Supplementary Figure 4. Maximum-likelihood evolutionary tree of NS sequences showing phylogenetic similarity of the H11 virus, A/Snowy sheathbill/Antarctica/2899/2014 (orange branch), detected in 2014, with the H11N2 viruses detected in Adélie penguins in 2013 (red branches).

Supplementary Figure 5. Maximum-likelihood evolutionary tree of 1,370 H5-HA sequences from avian, equine, human and swine viruses. Blue branches represent

North American avian lineages, whereas light green branches denote Eurasian avian lineages.

Supplementary Figure 6. Maximum-likelihood evolutionary tree of 405 N5-NA sequences from avian, equine, human and swine viruses. Blue branches represent North American avian lineages, whereas light green branches denote Eurasian avian lineages.

Supplementary Figure 7. Maximum-likelihood evolutionary tree of 2,102 PB2 sequences from avian, equine, human and swine viruses. Blue branches represent North American avian lineages, whereas light green branches denote Eurasian avian lineages.

Supplementary Figure 8. Maximum-likelihood evolutionary tree of 2,102 PB1 sequences from avian, equine, human and swine viruses. Blue branches represent North American avian lineages, whereas light green branches denote Eurasian avian lineages.

Supplementary Figure 9. Maximum-likelihood evolutionary tree of 2,102 PA sequences from avian, equine, human and swine viruses. Blue branches represent North American avian lineages, whereas light green branches denote Eurasian avian lineages.

Supplementary Figure 10. Maximum-likelihood evolutionary tree of 2,102 NP sequences from avian, equine, human and swine viruses. Blue branches represent North American avian lineages, whereas light green branches denote Eurasian avian lineages.

Supplementary Figure 11. Maximum-likelihood evolutionary tree of 2,102 MP sequences from avian, equine, human and swine viruses. Blue branches represent

North American avian lineages, whereas light green branches denote Eurasian avian lineages.

Supplementary Figure 12. Maximum-likelihood evolutionary tree of 2,102 NS sequences from avian, equine, human and swine viruses. Blue branches represent North American avian lineages, whereas light green branches denote Eurasian avian lineages.

Supplementary Figure 13. Dated evolutionary tree of H5-HA sequences from avian, equine, human and swine viruses. Blue branches represent North American avian lineages, whereas light green branches denote Eurasian avian lineages.

Supplementary Figure 14. Dated evolutionary tree of N5-NA sequences from avian, equine, human and swine viruses. Blue branches represent North American avian lineages, whereas light green branches denote Eurasian avian lineages.

Supplementary Figure 15. Dated evolutionary tree of PB2 sequences from avian, equine, human and swine viruses. Blue branches represent North American avian lineages, whereas light green branches denote Eurasian avian lineages.

Supplementary Figure 16. Dated evolutionary tree of PB1 sequences from avian, equine, human and swine viruses. Blue branches represent North American avian lineages, whereas light green branches denote Eurasian avian lineages.

Supplementary Figure 17. Dated evolutionary tree of PA sequences from avian, equine, human and swine viruses. Blue branches represent North American avian lineages, whereas light green branches denote Eurasian avian lineages.

Supplementary Figure 18. Dated evolutionary tree of NP sequences from avian, equine, human and swine viruses. Blue branches represent North American avian lineages, whereas light green branches denote Eurasian avian lineages.

Supplementary Figure 19. Dated evolutionary tree of MP sequences from avian, equine, human and swine viruses. Blue branches represent North American avian lineages, whereas light green branches denote Eurasian avian lineages.

Supplementary Figure 20. Dated evolutionary tree of NS sequences from avian, equine, human and swine viruses. Blue branches represent North American avian lineages, whereas light green branches denote Eurasian avian lineages.

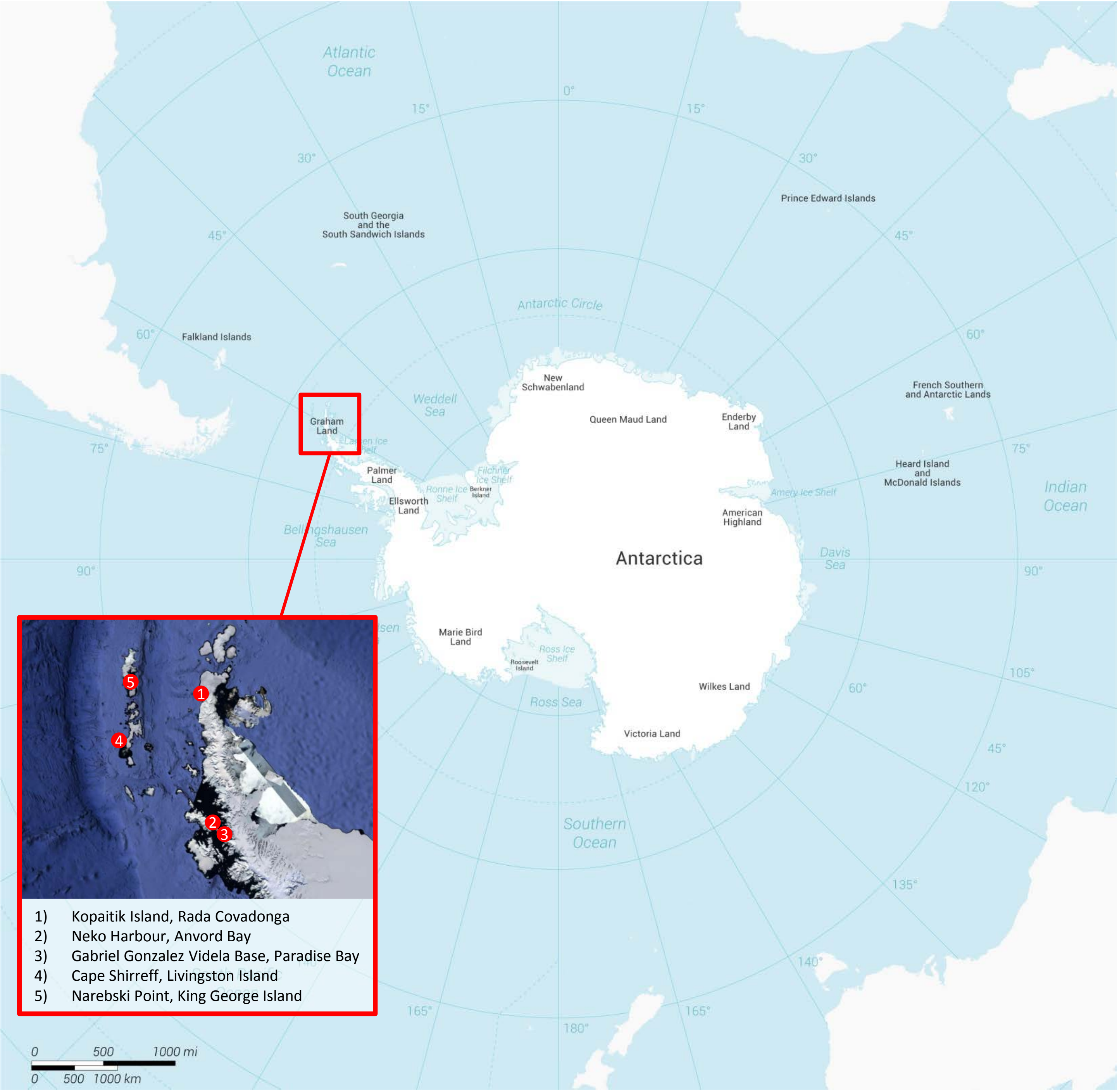

Atlantic Ocean
Indian Ocean
Southern Ocean
Antarctica
Antarctic Circle
South Georgia and the South Sandwich Islands
Falkland Islands
Prince Edward Islands
French Southern and Antarctic Lands
Heard Island and McDonald Islands
Graham Land
Larsen Ice Shelf
Palmer Land
Ellsworth Land
Weddell Sea
Ronne Ice Shelf
Filchner Ice Shelf
Berkner Island
Bellingshausen Sea
New Schwabenland
Queen Maud Land
Enderby Land
Amery Ice Shelf
American Highland
Davis Sea
Marie Bird Land
Ross Ice Shelf
Roosevelt Island
Ross Sea
Wilkes Land
Victoria Land
1) Kopaitik Island, Rada Covadonga
2) Neko Harbour, Anvord Bay
3) Gabriel Gonzalez Videla Base, Paradise Bay
4) Cape Shirreff, Livingston Island
5) Narebski Point, King George Island
0 500 1000 mi
0 500 1000 km

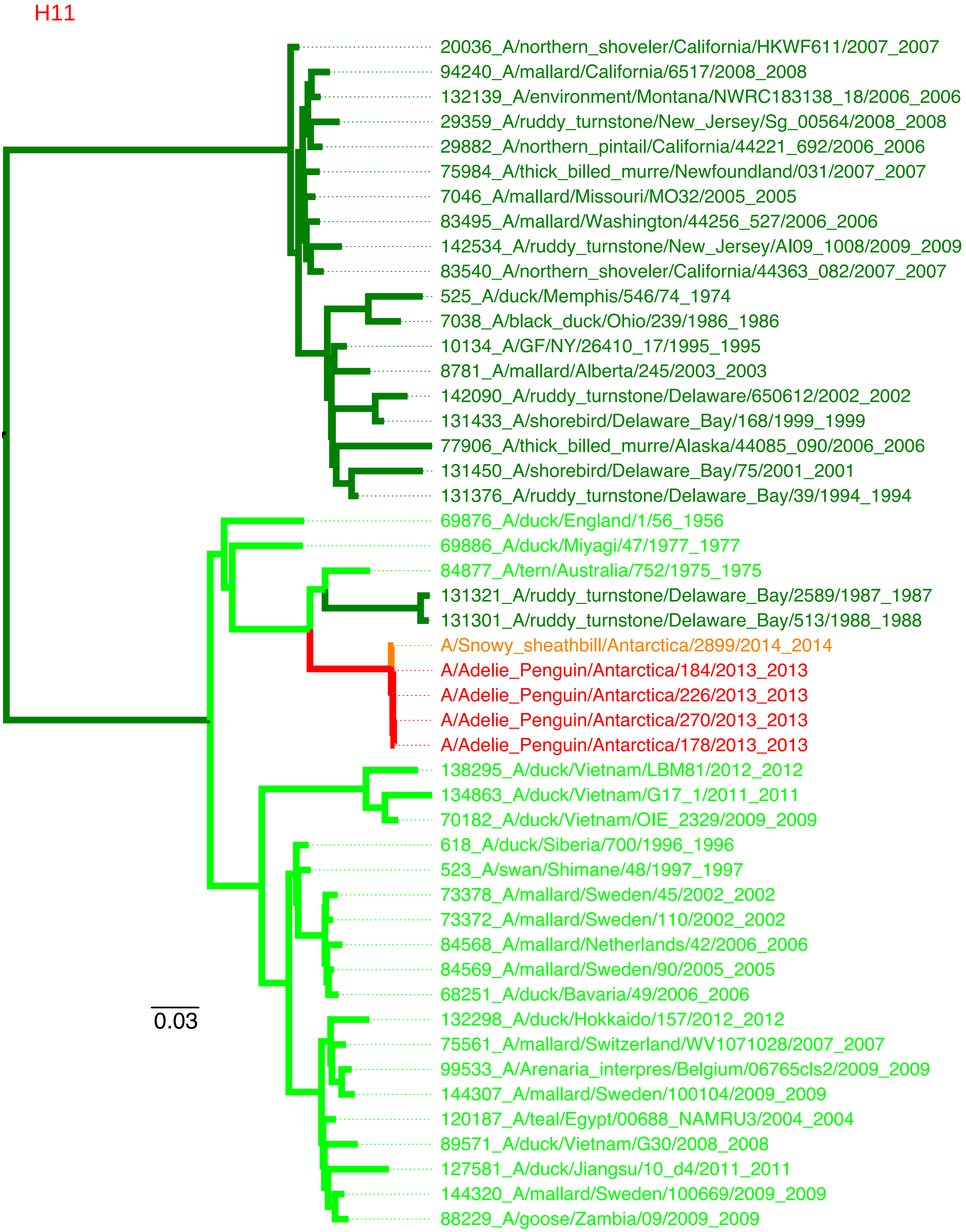
H11
20036_A/northern_shoveler/California/HKWF611/2007_2007
94240_A/mallard/California/6517/2008_2008
132139_A/environment/Montana/NWRC183138_18/2006_2006
29359_A/ruddy_turnstone/New_Jersey/Sg_00564/2008_2008
29882_A/northern_pintail/California/44221_692/2006_2006
75984_A/thick_billed_murre/Newfoundland/031/2007_2007
7046_A/mallard/Missouri/MO32/2005_2005
83495_A/mallard/Washington/44256_527/2006_2006
142534_A/ruddy_turnstone/New_Jersey/AI09_1008/2009_2009
83540_A/northern_shoveler/California/44363_082/2007_2007
525_A/duck/Memphis/546/74_1974
7038_A/black_duck/Ohio/239/1986_1986
10134_A/GF/NY/26410_17/1995_1995
8781_A/mallard/Alberta/245/2003_2003
142090_A/ruddy_turnstone/Delaware/650612/2002_2002
131433_A/shorebird/Delaware_Bay/168/1999_1999
77906_A/thick_billed_murre/Alaska/44085_090/2006_2006
131450_A/shorebird/Delaware_Bay/75/2001_2001
131376_A/ruddy_turnstone/Delaware_Bay/39/1994_1994
69876_A/duck/England/1/56_1956
69886_A/duck/Miyagi/47/1977_1977
84877_A/tern/Australia/752/1975_1975
131321_A/ruddy_turnstone/Delaware_Bay/2589/1987_1987
131301_A/ruddy_turnstone/Delaware_Bay/513/1988_1988
A/Snowy_sheathbill/Antarctica/2899/2014_2014
A/Adelie_Penguin/Antarctica/184/2013_2013
A/Adelie_Penguin/Antarctica/226/2013_2013
A/Adelie_Penguin/Antarctica/270/2013_2013
A/Adelie_Penguin/Antarctica/178/2013_2013
138295_A/duck/Vietnam/LBM81/2012_2012
134863_A/duck/Vietnam/G17_1/2011_2011
70182_A/duck/Vietnam/OIE_2329/2009_2009
618_A/duck/Siberia/700/1996_1996
523_A/swan/Shimane/48/1997_1997
73378_A/mallard/Sweden/45/2002_2002
73372_A/mallard/Sweden/110/2002_2002
84568_A/mallard/Netherlands/42/2006_2006
84569_A/mallard/Sweden/90/2005_2005
68251_A/duck/Bavaria/49/2006_2006
132298_A/duck/Hokkaido/157/2012_2012
75561_A/mallard/Switzerland/WV1071028/2007_2007
99533_A/Arenaria_interpres/Belgium/06765cls2/2009_2009
144307_A/mallard/Sweden/100104/2009_2009
120187_A/teal/Egypt/00688_NAMRU3/2004_2004
89571_A/duck/Vietnam/G30/2008_2008
127581_A/duck/Jiangsu/10_d4/2011_2011
144320_A/mallard/Sweden/100669/2009_2009
88229_A/goose/Zambia/09/2009_2009
0.03

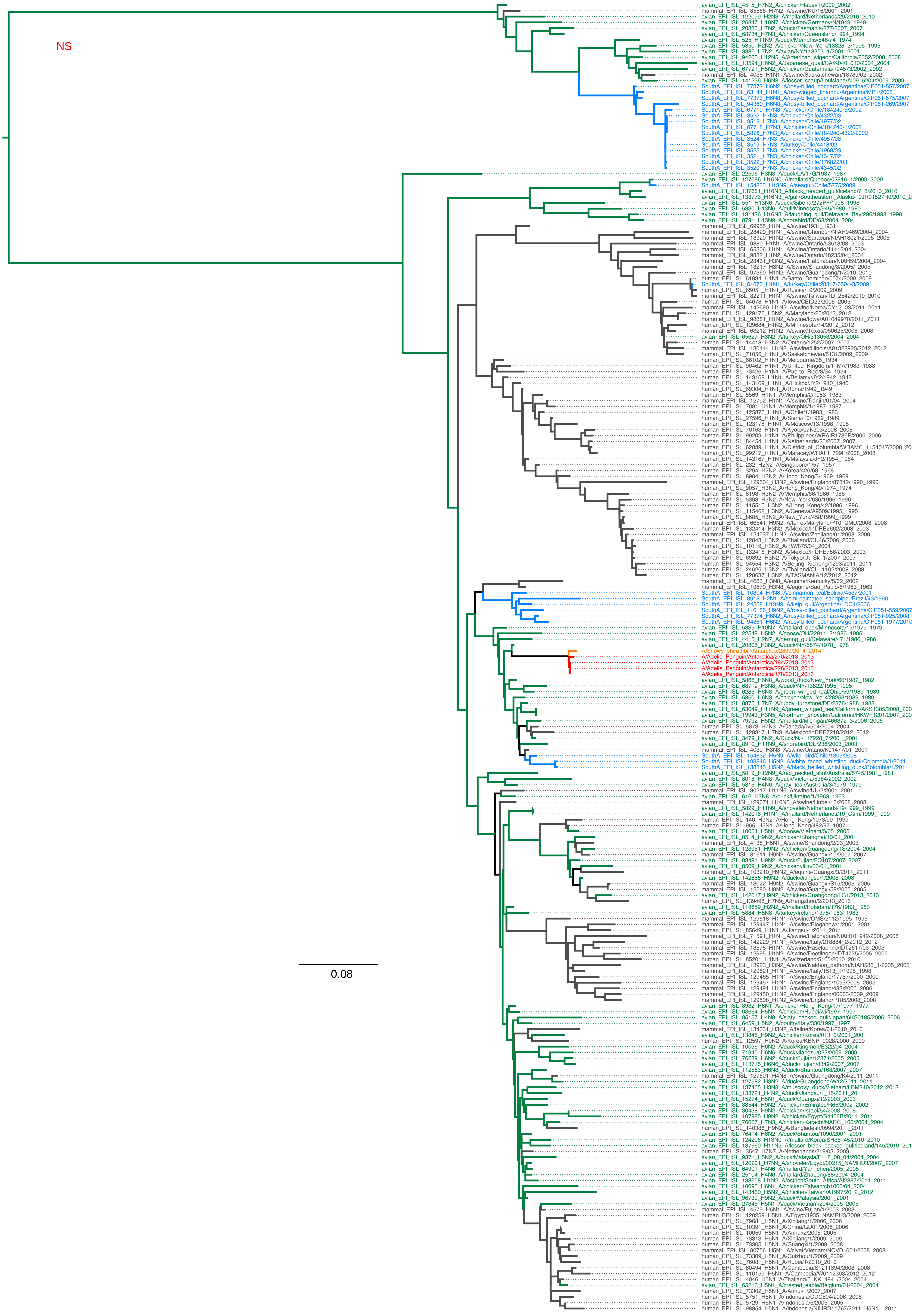

NS
0.08

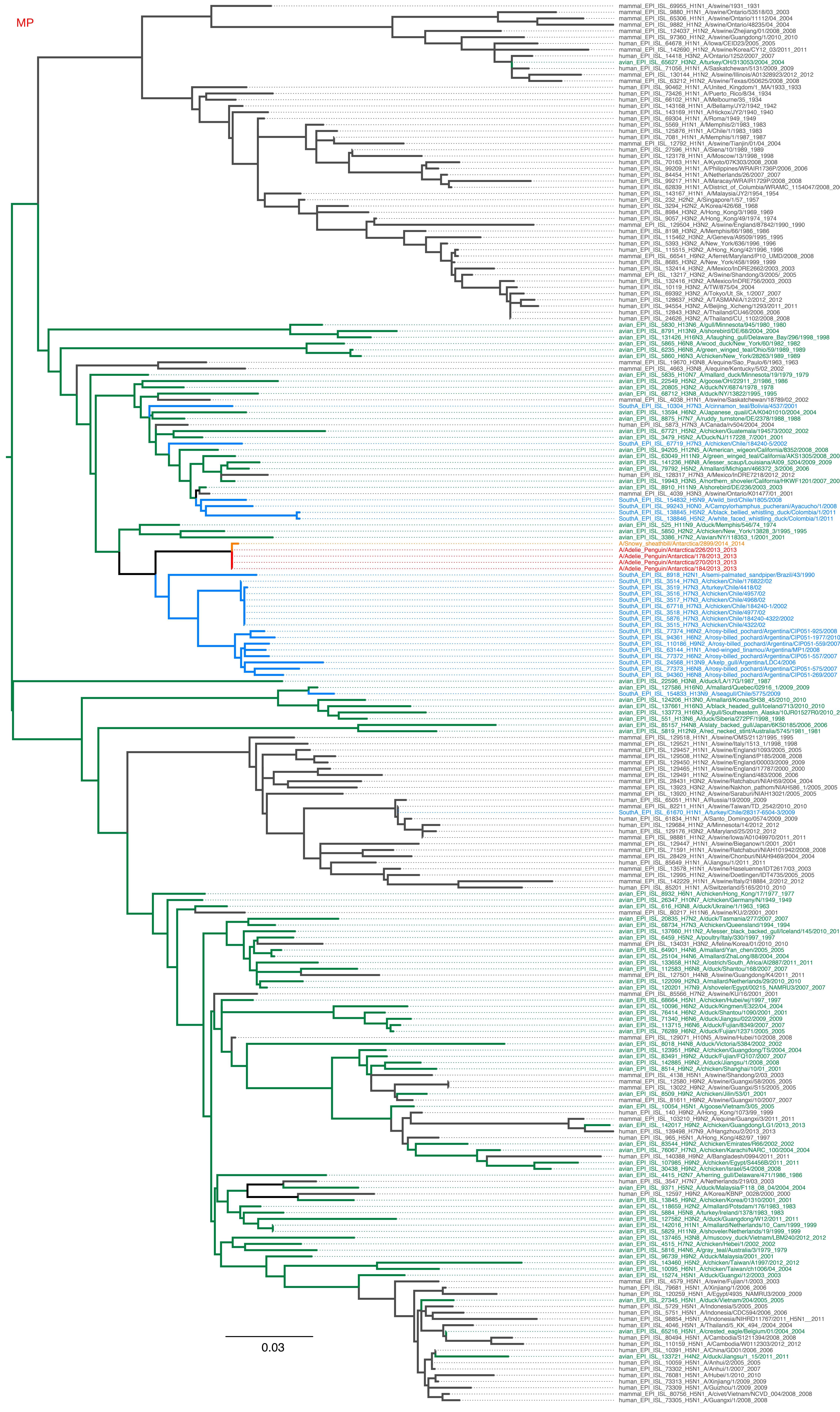
MP
0.03

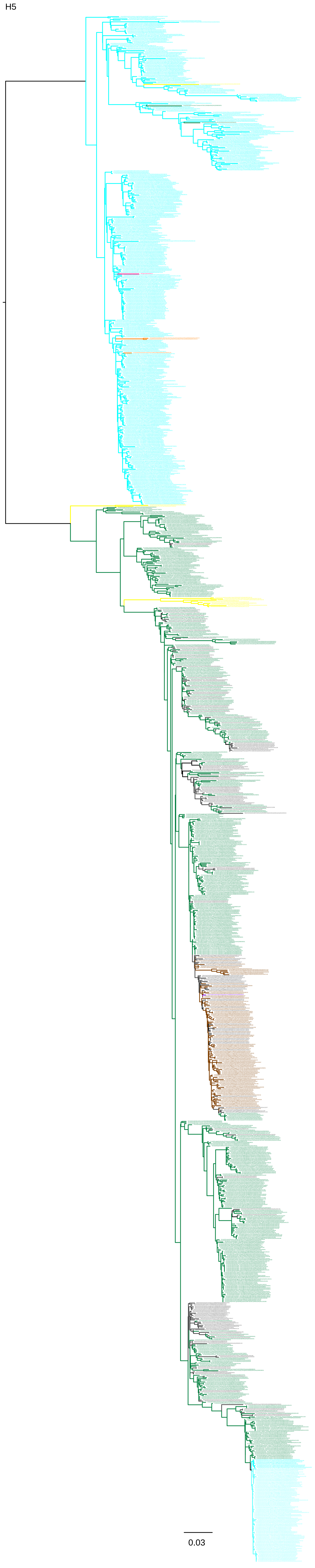
H5
0.03

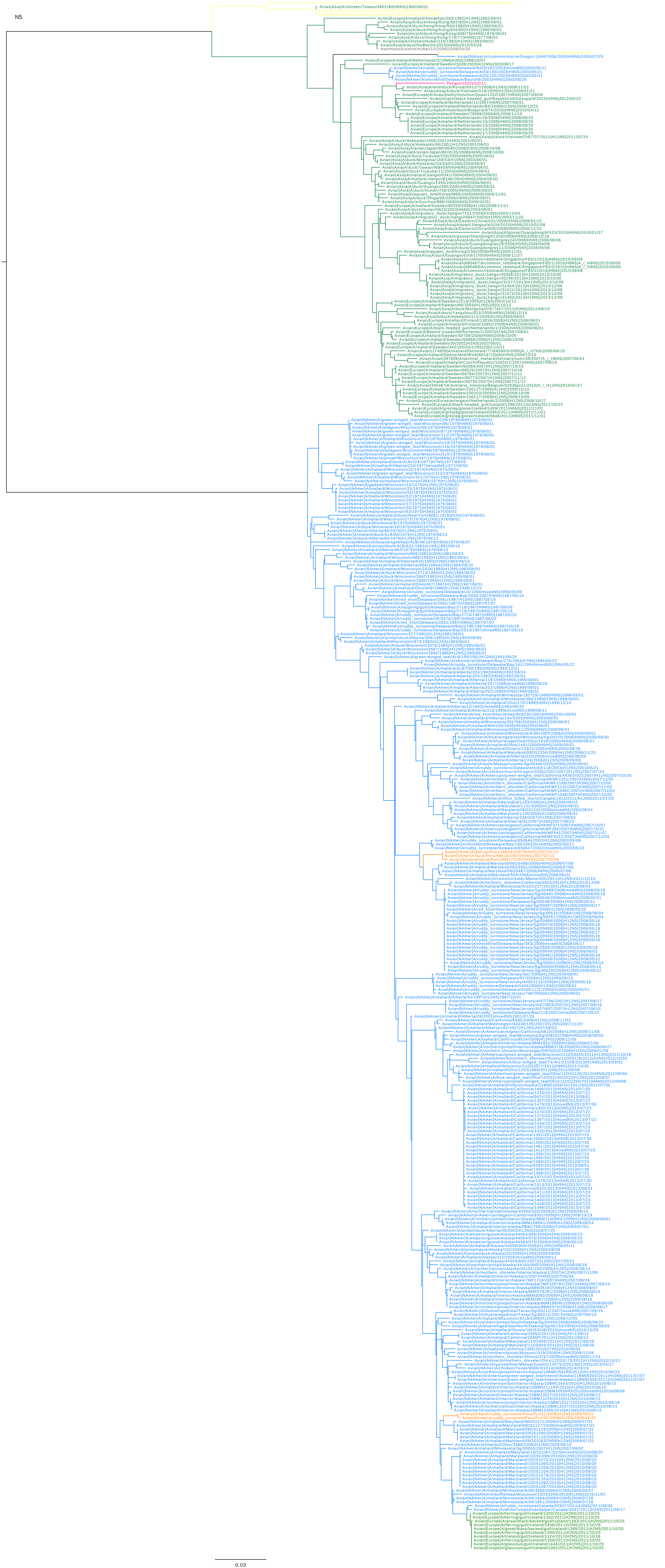
N5
0.03

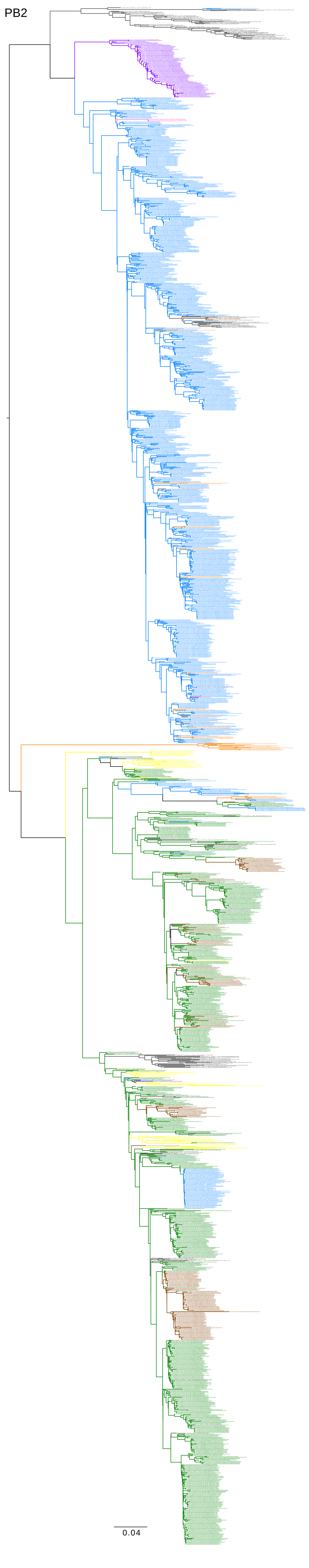
PB2
0.04

PB1

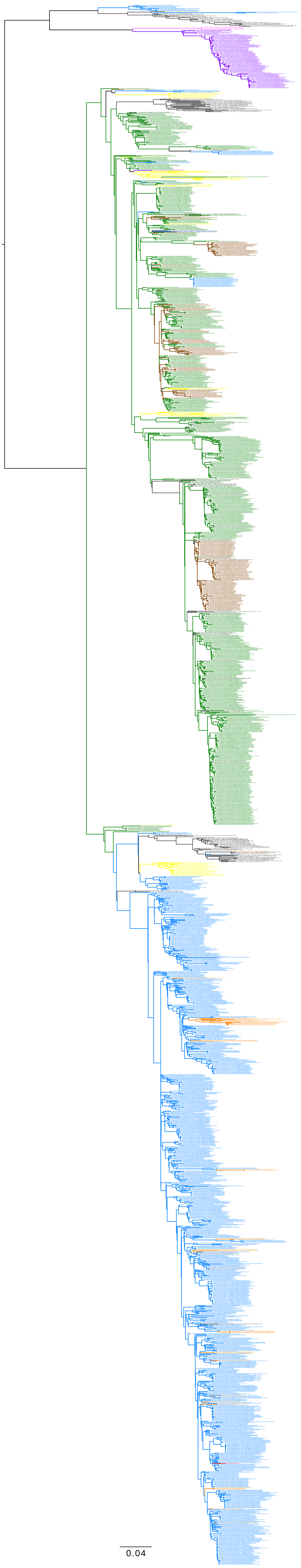

PA

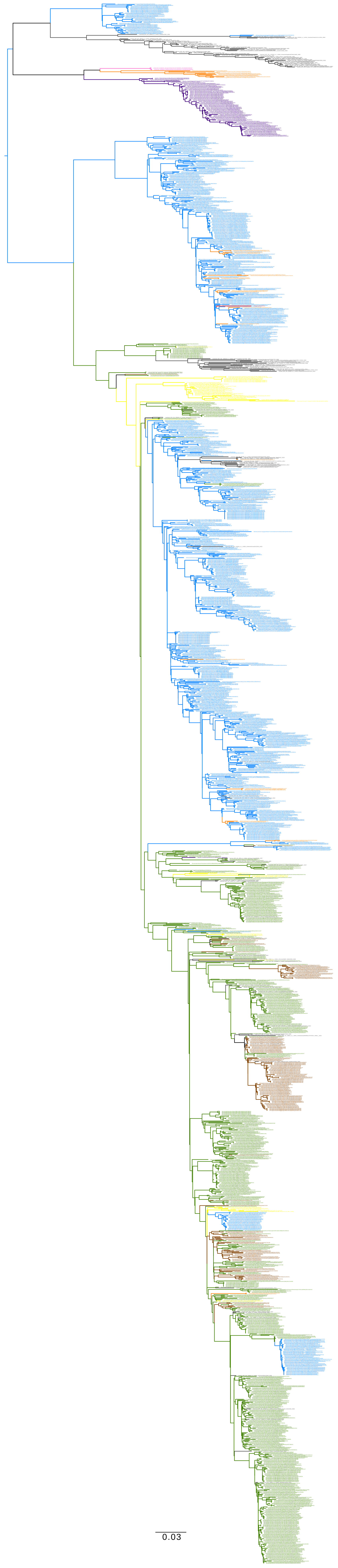

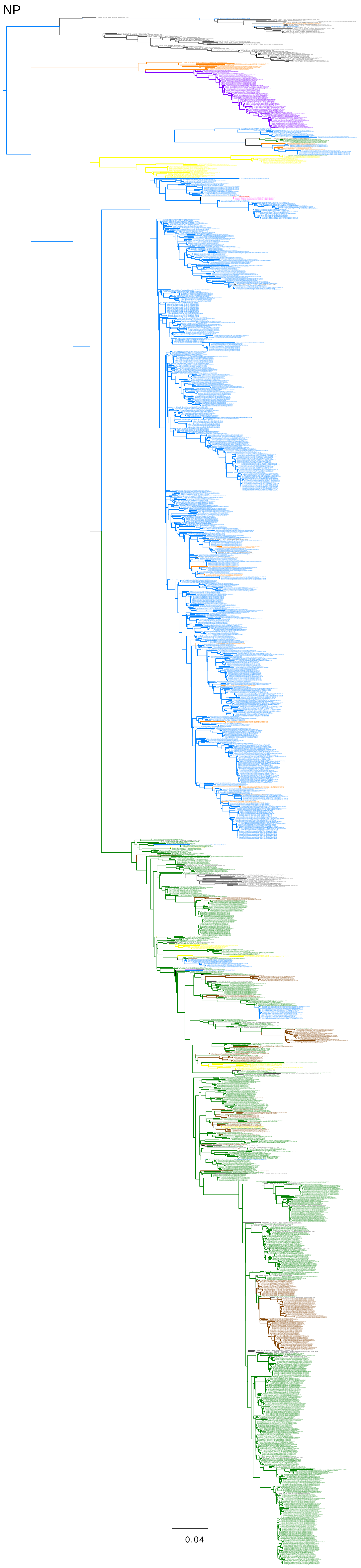
NP
0.04

MP

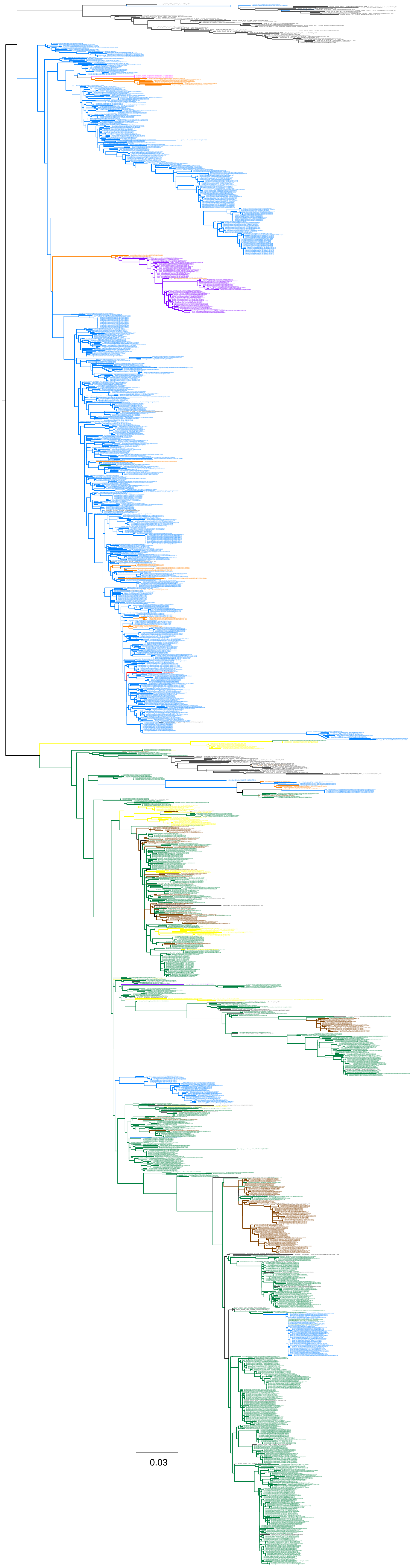

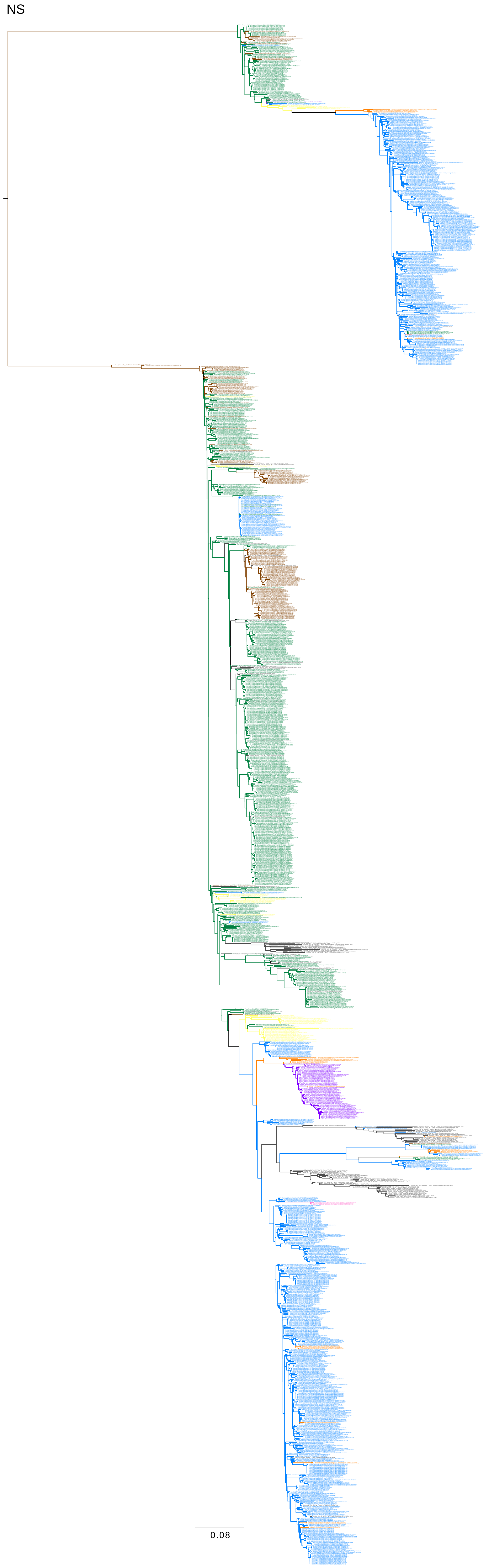
NS
0.08

H5

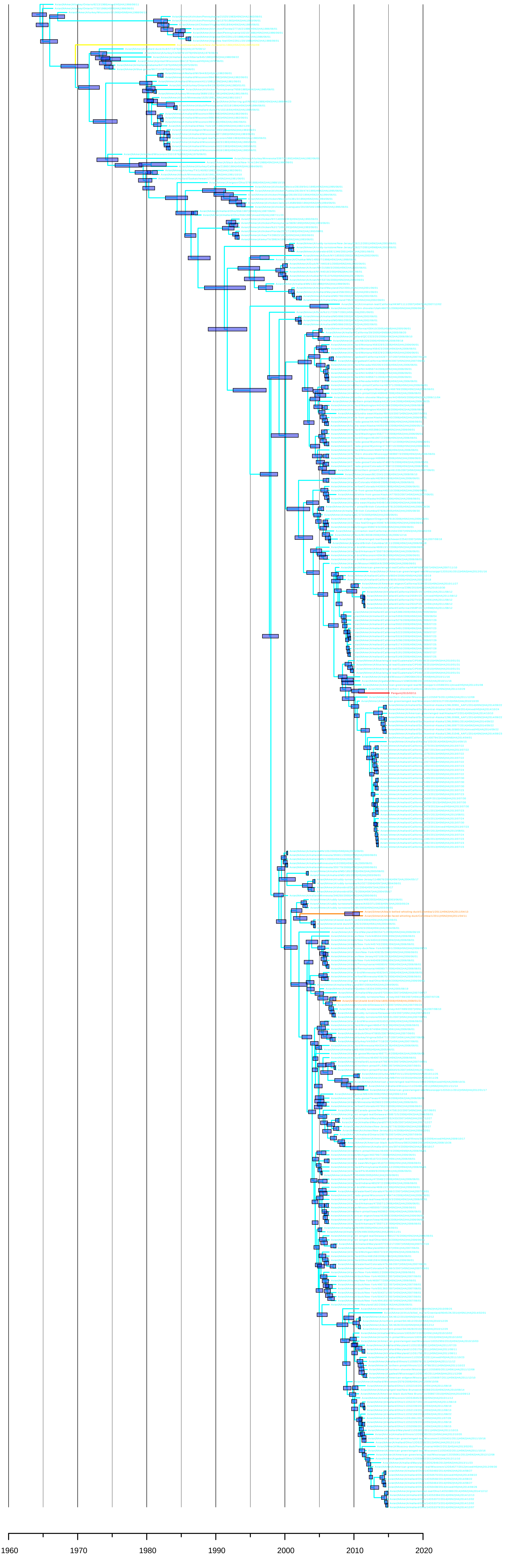

N5

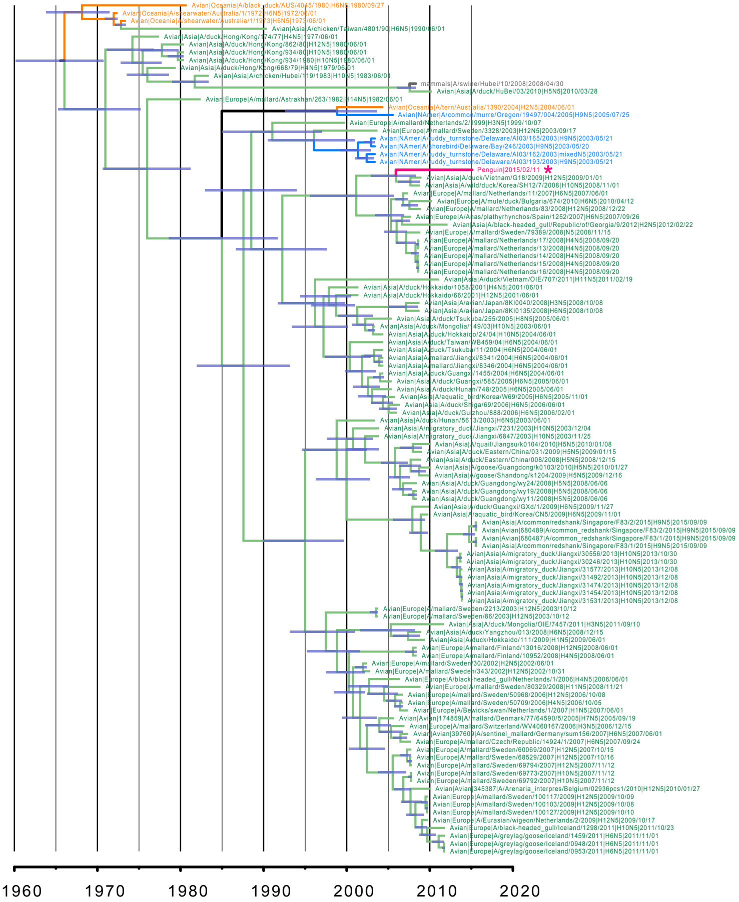

Avian|Oceania|A/black_duck/AUS/4045/1980|H6N5|1980/09/27
Avian|Oceania|A/shearwater/Australia/1/1972|H6N5|1972/06/01
Avian|Oceania|A/shearwater/Australia/1/1973|H6N5|1973/06/01
Avian|Asia|A/chicken/Taiwan/4801/90|H6N5|1990/06/01
Avian|Asia|A/duck/Hong/Kong/174/77|H4N5|1977/06/01
Avian|Asia|A/duck/Hong/Kong/862/80|H12N5|1980/06/01
Avian|Asia|A/duck/Hong/Kong/934/80|H10N5|1980/06/01
Avian|Asia|A/duck/Hong/Kong/934/1980|H10N5|1980/06/01
Avian|Asia|A/duck/Hong/Kong/668/79|H4N5|1979/06/01
Avian|Asia|A/chicken/Hubei/119/1983|H10N5|1983/06/01
mammals|A/swine/Hubei/10/2008|2008/04/30
Avian|Asia|A/duck/HuBei/03/2010|H5N5|2010/03/28
Avian|Europe|A/mallard/Astrakhan/263/1982|H14N5|1982/06/01
Avian|Oceania|A/tern/Australia/1390/2004|H2N5|2004/06/01
Avian|NAmer|A/common/murre/Oregon/19497/004/2005|H9N5|2005/07/25
Avian|Europe|A/mallard/Netherlands/2/1999|H3N5|1999/10/07
Avian|Europe|A/mallard/Sweden/3328/2003|H12N5|2003/09/17
Avian|NAmer|A/ruddy_turnstone/Delaware/AI03/165/2003|H9N5|2003/05/21
Avian|NAmer|A/shorebird/Delaware/Bay/246/2003|H9N5|2003/05/20
Avian|NAmer|A/ruddy_turnstone/Delaware/AI03/162/2003|mixedN5|2003/05/21
Avian|NAmer|A/ruddy_turnstone/Delaware/AI03/193/2003|H9N5|2003/05/21
Penguin|2015/02/11 *
Avian|Asia|A/duck/Vietnam/G18/2009|H12N5|2009/01/01
Avian|Asia|A/wild/duck/Korea/SH12/7/2008|H10N5|2008/11/01
Avian|Europe|A/mallard/Netherlands/11/2007|H6N5|2007/06/01
Avian|Europe|A/mule/duck/Bulgaria/674/2010|H6N5|2010/04/12
Avian|Europe|A/mallard/Netherlands/83/2008|H12N5|2008/12/22
Avian|Europe|A/Anas/platyrhynchos/Spain/1252/2007|H6N5|2007/09/26
Avian|Asia|A/black-headed_gull/Republic/of/Georgia/9/2012|H2N5|2012/02/22
Avian|Europe|A/mallard/Sweden/79389/2008|N5|2008/11/15
Avian|Europe|A/mallard/Netherlands/17/2008|H4N5|2008/09/20
Avian|Europe|A/mallard/Netherlands/13/2008|H4N5|2008/09/20
Avian|Europe|A/mallard/Netherlands/14/2008|H4N5|2008/09/20
Avian|Europe|A/mallard/Netherlands/15/2008|H4N5|2008/09/20
Avian|Europe|A/mallard/Netherlands/16/2008|H4N5|2008/09/20
Avian|Asia|A/duck/Vietnam/OIE/707/2011|H11N5|2011/02/19
Avian|Asia|A/duck/Hokkaido/1058/2001|H4N5|2001/06/01
Avian|Asia|A/duck/Hokkaido/66/2001|H12N5|2001/06/01
Avian|Asia|A/avian/Japan/8KI0040/2008|H3N5|2008/10/08
Avian|Asia|A/avian/Japan/8KI0135/2008|H6N5|2008/10/08
Avian|Asia|A/duck/Tsukuba/255/2005|H8N5|2005/06/01
Avian|Asia|A/duck/Mongolia/149/03|H10N5|2003/06/01
Avian|Asia|A/duck/Hokkaido/24/04|H10N5|2004/06/01
Avian|Asia|A/duck/Taiwan/WB459/04|H6N5|2004/06/01
Avian|Asia|A/duck/Tsukuba/11/2004|H6N5|2004/06/01
Avian|Asia|A/mallard/Jiangxi/8341/2004|H6N5|2004/06/01
Avian|Asia|A/mallard/Jiangxi/8346/2004|H6N5|2004/06/01
Avian|Asia|A/duck/Guangxi/1455/2004|H6N5|2004/06/01
Avian|Asia|A/duck/Guangxi/585/2005|H6N5|2005/06/01
Avian|Asia|A/duck/Hunan/748/2005|H6N5|2005/06/01
Avian|Asia|A/aquatic_bird/Korea/W69/2005|H6N5|2005/11/01
Avian|Asia|A/duck/Shiga/69/2006|H6N5|2006/06/01
Avian|Asia|A/duck/Guizhou/888/2006|H6N5|2006/02/01
Avian|Asia|A/duck/Hunan/5613/2003|H6N5|2003/06/01
Avian|Asia|A/migratory_duck/Jiangxi/7231/2003|H10N5|2003/12/04
Avian|Asia|A/migratory_duck/Jiangxi/6847/2003|H10N5|2003/11/25
Avian|Asia|A/quail/Jiangsu/k0104/2010|H5N5|2010/01/08
Avian|Asia|A/duck/Eastern/China/031/2009|H5N5|2009/01/15
Avian|Asia|A/duck/Eastern/China/008/2008|H5N5|2008/12/15
Avian|Asia|A/goose/Guangdong/k0103/2010|H5N5|2010/01/27
Avian|Asia|A/goose/Shandong/k1204/2009|H5N5|2009/12/16
Avian|Asia|A/duck/Guangdong/wy24/2008|H5N5|2008/06/06
Avian|Asia|A/duck/Guangdong/wy19/2008|H5N5|2008/06/06
Avian|Asia|A/duck/Guangdong/wy11/2008|H5N5|2008/06/06
Avian|Asia|A/duck/Guangxi/GXd/1/2009|H6N5|2009/11/27
Avian|Asia|A/aquatic_bird/Korea/CN5/2009|H6N5|2009/11/01
Avian|Asia|A/common/redshank/Singapore/F83/2/2015|H9N5|2015/09/09
Avian|Avian|680489|A/common_redshank/Singapore/F83/2/2015|H9N5|2015/09/09
Avian|Avian|680487|A/common_redshank/Singapore/F83/1/2015|H9N5|2015/09/09
Avian|Asia|A/common/redshank/Singapore/F83/1/2015|H9N5|2015/09/09
Avian|Asia|A/migratory_duck/Jiangxi/30556/2013|H10N5|2013/10/30
Avian|Asia|A/migratory_duck/Jiangxi/30246/2013|H10N5|2013/10/30
Avian|Asia|A/migratory_duck/Jiangxi/31577/2013|H10N5|2013/12/08
Avian|Asia|A/migratory_duck/Jiangxi/31492/2013|H10N5|2013/12/08
Avian|Asia|A/migratory_duck/Jiangxi/31474/2013|H10N5|2013/12/08
Avian|Asia|A/migratory_duck/Jiangxi/31454/2013|H10N5|2013/12/08
Avian|Asia|A/migratory_duck/Jiangxi/31531/2013|H10N5|2013/12/08
Avian|Europe|A/mallard/Sweden/2213/2003|H12N5|2003/10/12
Avian|Europe|A/mallard/Sweden/86/2003|H12N5|2003/10/12
Avian|Asia|A/duck/Mongolia/OIE/7457/2011|H3N5|2011/09/10
Avian|Asia|A/duck/Yangzhou/013/2008|H6N5|2008/12/15
Avian|Asia|A/duck/Hokkaido/111/2009|H1N5|2009/06/01
Avian|Europe|A/mallard/Finland/13016/2008|H12N5|2008/06/01
Avian|Europe|A/mallard/Finland/10952/2008|H4N5|2008/06/01
Avian|Europe|A/mallard/Sweden/30/2002|H2N5|2002/06/01
Avian|Europe|A/mallard/Sweden/343/2002|H12N5|2002/10/31
Avian|Europe|A/black-headed_gull/Netherlands/1/2006|H4N5|2006/06/01
Avian|Europe|A/mallard/Sweden/80329/2008|H11N5|2008/11/21
Avian|Europe|A/mallard/Sweden/50968/2006|H12N5|2006/10/08
Avian|Europe|A/mallard/Sweden/50709/2006|H4N5|2006/10/05
Avian|Europe|A/Bewicks/swan/Netherlands/1/2007|H1N5|2007/06/01
Avian|Avian|174859|A/mallard/Denmark/77/64590/5/2005|H7N5|2005/09/19
Avian|Europe|A/mallard/Switzerland/WV4060167/2006|H3N5|2006/12/15
Avian|Avian|397609|A/sentinel_mallard/Germany/sum156/2007|H6N5|2007/06/01
Avian|Europe|A/mallard/Czech/Republic/14924/1/2007|H6N5|2007/09/24
Avian|Europe|A/mallard/Sweden/60069/2007|H12N5|2007/10/15
Avian|Europe|A/mallard/Sweden/68529/2007|H12N5|2007/10/16
Avian|Europe|A/mallard/Sweden/69794/2007|H12N5|2007/11/12
Avian|Europe|A/mallard/Sweden/69773/2007|H10N5|2007/11/12
Avian|Europe|A/mallard/Sweden/69792/2007|H10N5|2007/11/12
Avian|Avian|345387|A/Arenaria_interpres/Belgium/02936pcs1/2010|H12N5|2010/01/27
Avian|Europe|A/mallard/Sweden/100117/2009|H12N5|2009/10/09
Avian|Europe|A/mallard/Sweden/100103/2009|H12N5|2009/10/08
Avian|Europe|A/mallard/Sweden/100127/2009|H12N5|2009/10/10
Avian|Europe|A/Eurasian/wigeon/Netherlands/2/2009|H12N5|2009/10/17
Avian|Europe|A/black-headed_gull/Iceland/1298/2011|H10N5|2011/10/23
Avian|Europe|A/greylag/goose/Iceland/1459/2011|H6N5|2011/11/01
Avian|Europe|A/greylag/goose/Iceland/0948/2011|H6N5|2011/11/01
Avian|Europe|A/greylag/goose/Iceland/0953/2011|H6N5|2011/11/01
1960
1970
1980
1990
2000
2010
2020

PB2 (penguin H11N2 and novel penguin H5N5)

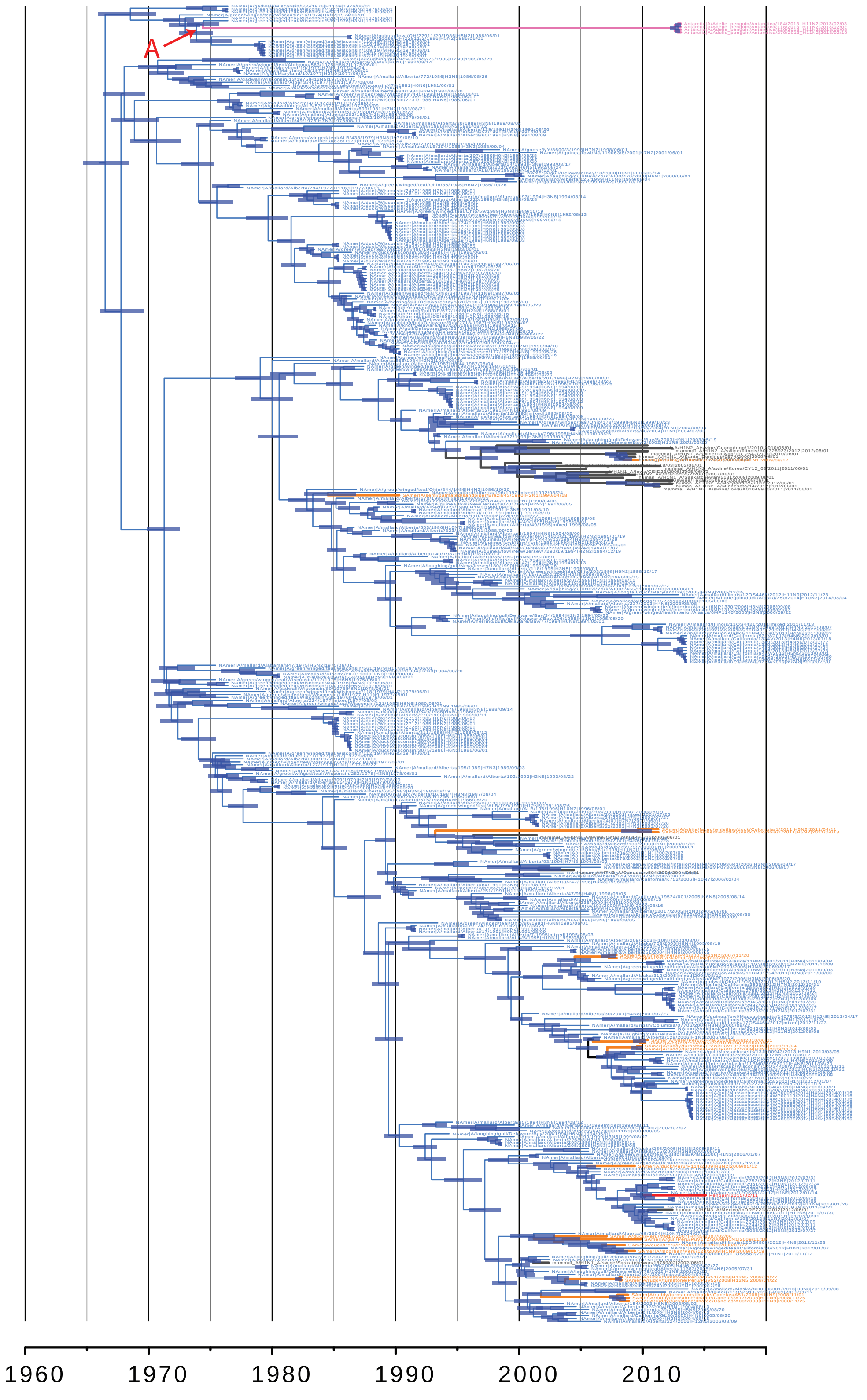

PB1 (penguin H11N2)

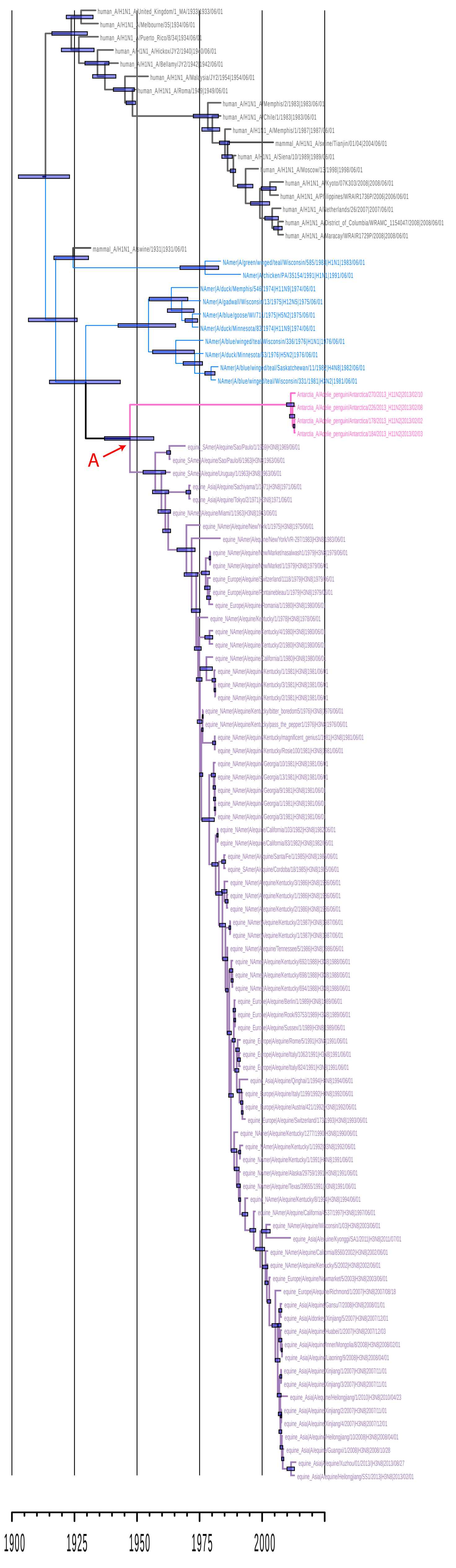

PB1 (novel penguin H5N5)

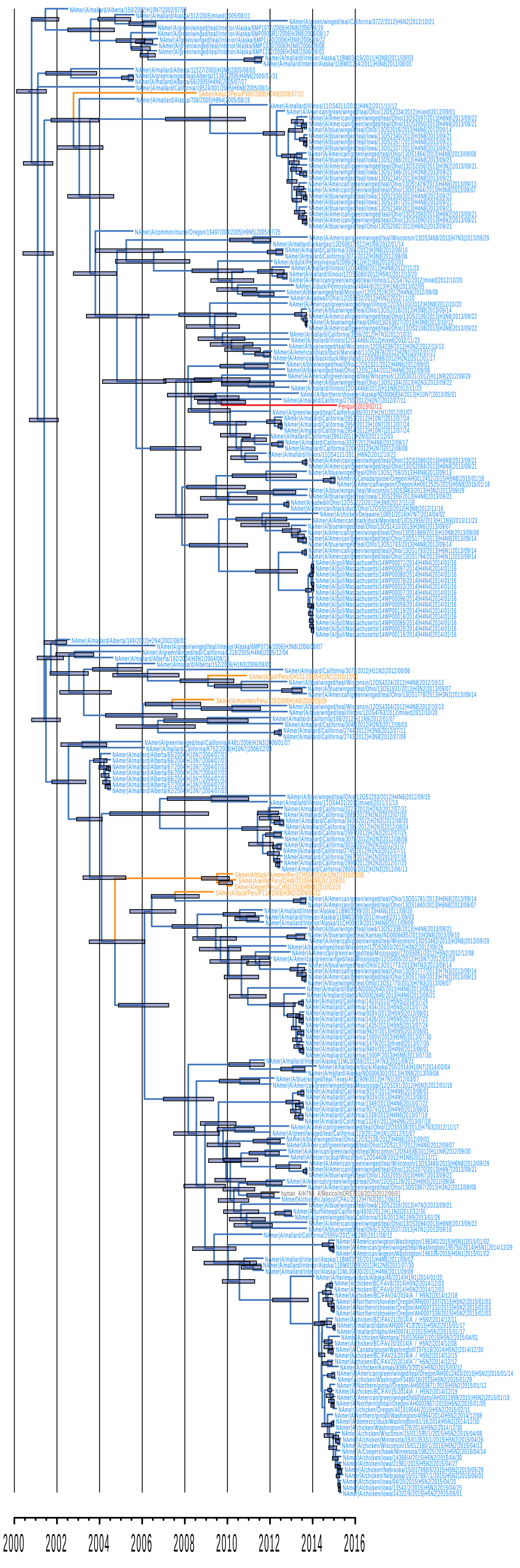

PA (Antarctica H11N2 lineage)

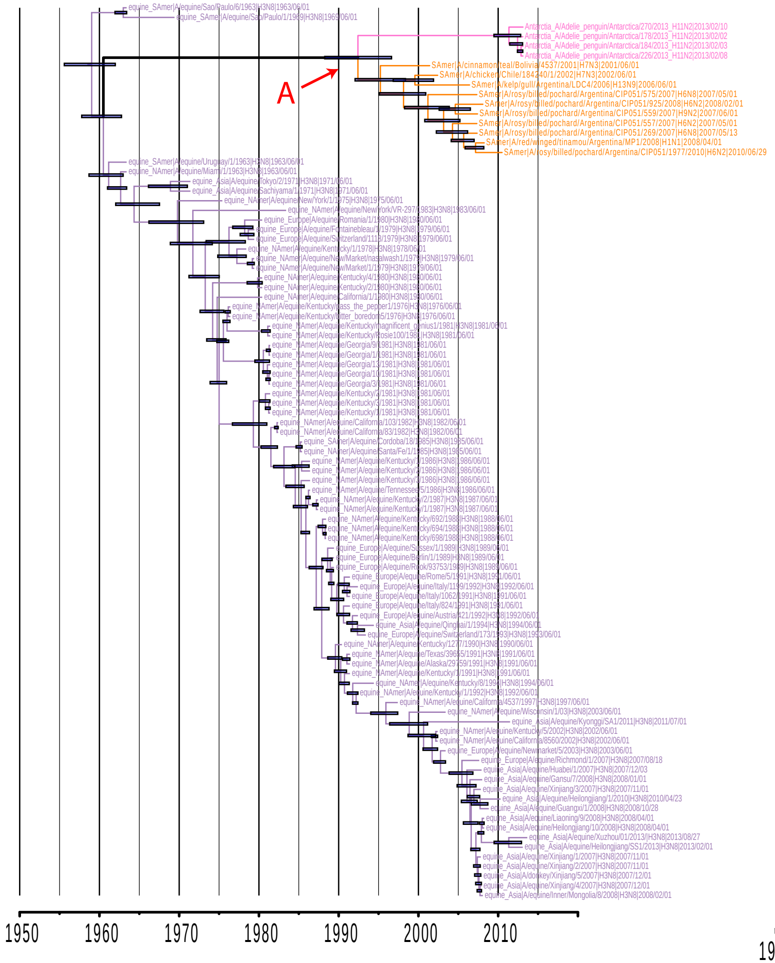

PA (new penguin H5N5)

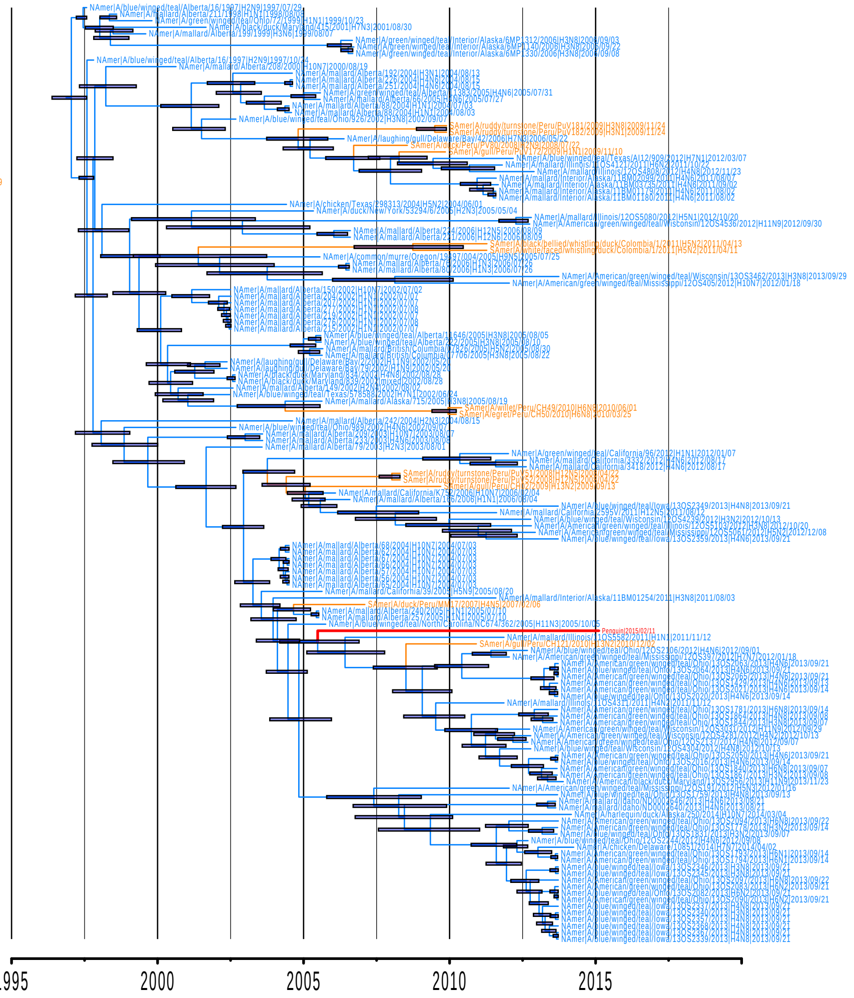

NP (penguin H11N2 and novel penguin H5N5)

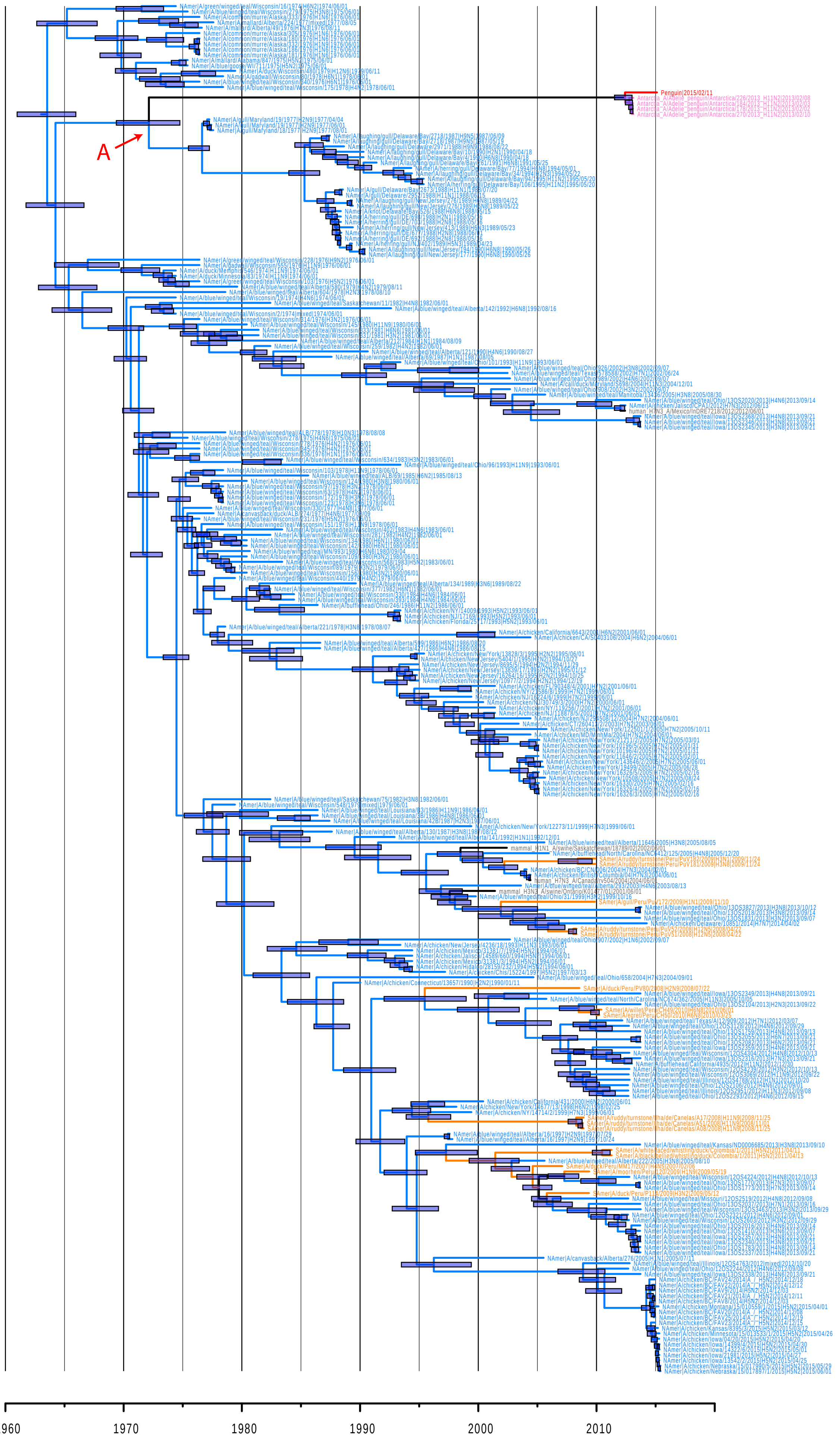

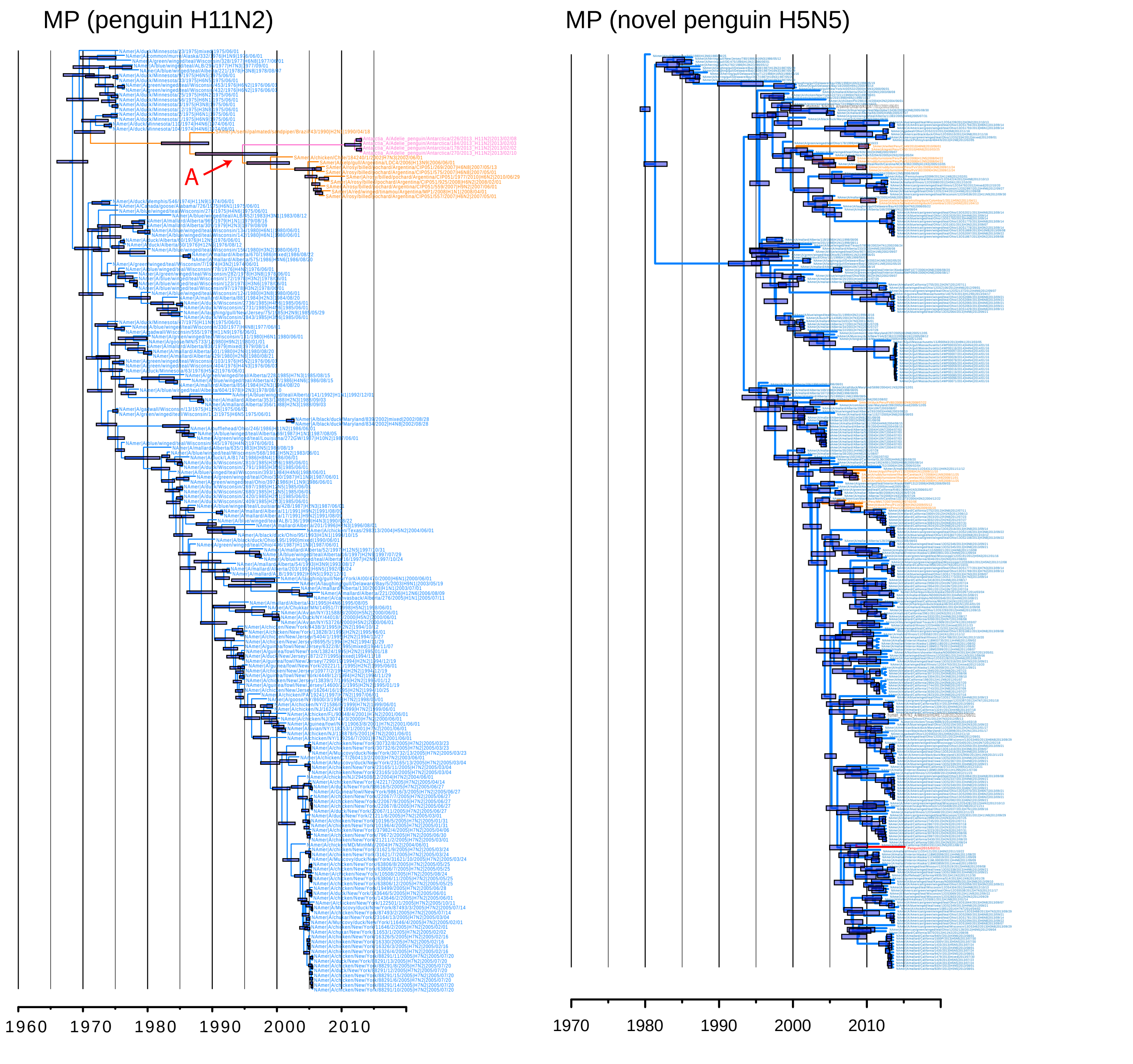

MP (penguin H11N2)
MP (novel penguin H5N5)
A
1960
1970
1980
1990
2000
2010
1970
1980
1990
2000
2010

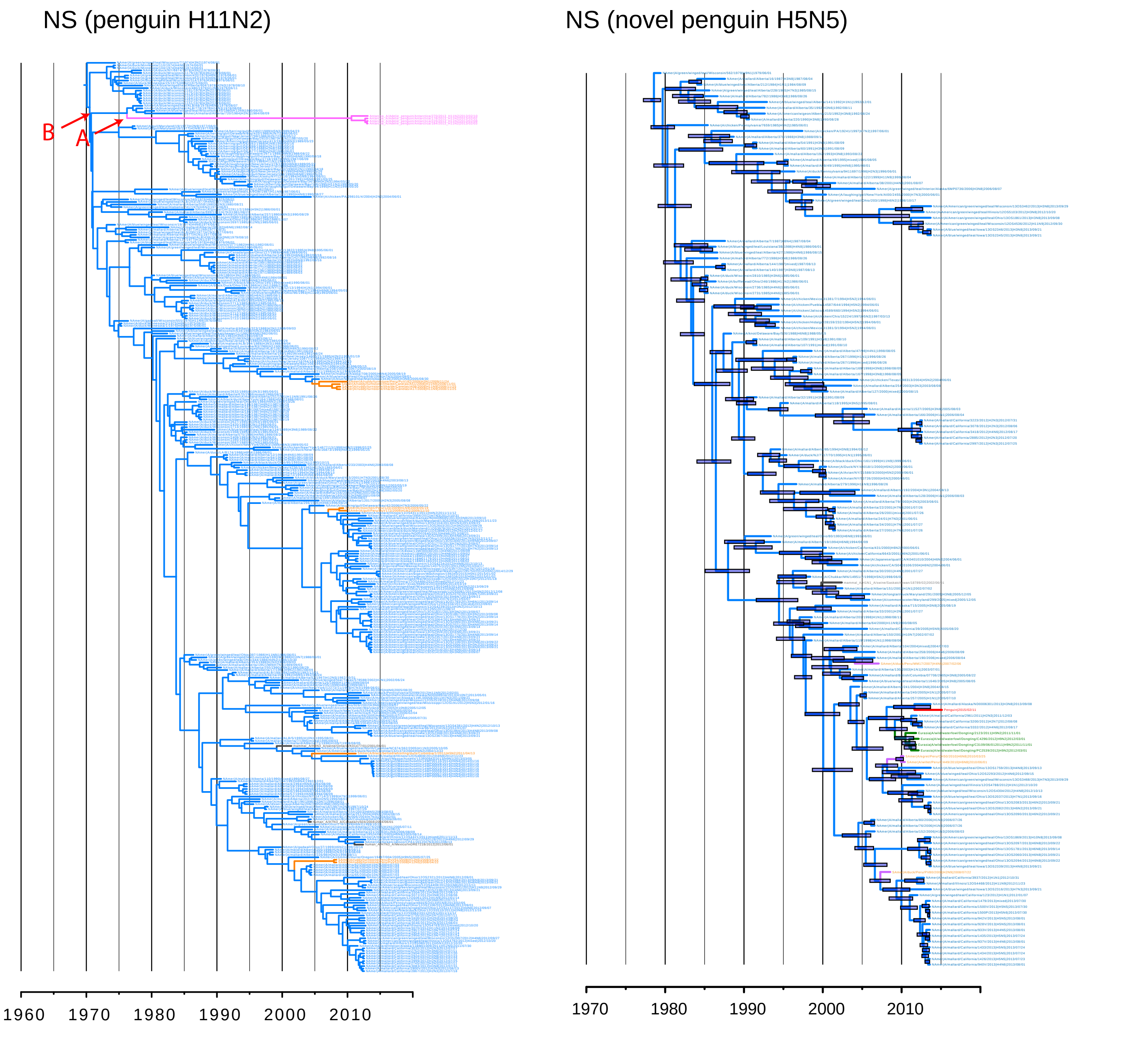

NS (penguin H11N2)
NS (novel penguin H5N5)
B
A
1960
1970
1980
1990
2000
2010
1970
1980
1990
2000
2010
